# Cytotoxic ROS-Consuming Mn(III) Synzymes: Structural Influence on Their Mechanism of Action

**DOI:** 10.3390/ijms26010150

**Published:** 2024-12-27

**Authors:** Lorenzo Verderi, Niccolò Nova, Valentina Borghesani, Matteo Tegoni, Marco Giannetto, Simone Fortunati, Luca Ronda, Silvana Pinelli, Paola Mozzoni, Maria Nicastro, Benedetta Ghezzi, Giorgio Pelosi, Franco Bisceglie

**Affiliations:** 1 Department of Chemistry, Life Sciences and Environmental Sustainability, University of Parma, 43124 Parma, Italy; lorenzo.verderi@unipr.it (L.V.); niccolo.nova@studenti.unipr.it (N.N.); valentina.borghesani@unipr.it (V.B.); matteo.tegoni@unipr.it (M.T.); marco.giannetto@unipr.it (M.G.); simone.fortunati@unipr.it (S.F.); 2 Department of Medicine and Surgery, University of Parma, Via Volturno, 39, 43125 Parma, Italy; luca.ronda@unipr.it (L.R.); silvana.pinelli@unipr.it (S.P.); paola.mozzoni@unipr.it (P.M.); maria.nicastro@unipr.it (M.N.); benedetta.ghezzi@unipr.it (B.G.); 3 Centre of Excellence for Toxicological Research (CERT), University of Parma, 43124 Parma, Italy

**Keywords:** Mn(III) complexes, synzymes, ROS, cytotoxicity

## Abstract

ROS (i.e., reactive oxygen species) scavenging is a key function of various Mn-based enzymes, including superoxide dismutases (SODs) and catalases, which are actively linked to oxidative stress-related diseases. In this study, we synthesized and characterized two novel Mn(III)-based synzymes (i.e., synthetic enzymes), designated **C1** ([Mn**L1**Cl(H_2_O)]Cl·3H_2_O) and **C2** ([Mn**L2**Cl_2_]·2H_2_O), which differ in the presence of a bridging aliphatic or aromatic group in the chelator. Using a range of analytical techniques, we found that the aromatic **C2** bridge significantly influences the Mn(III) center’s *cis-β* configuration, unlike **C1**, which adopts a *trans* configuration. We then thoroughly evaluated the oxidation-reduction properties of **C1** and **C2**, including their redox potentials (by cyclic voltammetry) and capacity to consume various ROS species (using DPPH, hydroxyl radical, hydrogen peroxide, and superoxide UV–visible spectrophotometric assays). The specific kinetics of the H_2_O_2_ dismutation process, as measured by a Clark-type electrode and time-resolved ESI-MS, revealed that both synzymes possess catalytic activity. Toxicological experiments using the *Galleria mellonella* larval model demonstrated the compounds’ innocuous nature towards higher eukaryotic organisms, while cytotoxicity assays confirmed their selective efficacy against lung cancer cells. Additional cytological assays, such as the thiobarbituric acid reactive substances assay and caspase-3 activity and p53 expression analysis, reported that **C1** and **C2** induce cytotoxicity against cancer cells via apoptosis rather than necrosis and behave very differently towards redox substances and ROS-regulating enzymes in vivo. These findings suggest that the structural differences between **C1** and **C2** lead to distinct redox properties and biological activities, highlighting the potential of these novel Mn(III)-based synzymes as therapeutic agents for the treatment of oxidative stress-related diseases, particularly lung cancer. Further studies are warranted to elucidate the underlying mechanisms of action and explore their clinical applications.

## 1. Introduction

Reactive oxygen species (ROS) are oxygen derivatives that are more reactive than molecular oxygen itself. They include free radicals and oxidants, which can be generated endogenously or triggered by external stimuli. ROS include both radical and non-radical molecules, such as superoxide, hydroxyl radicals, hydrogen peroxide, and ozone, which are produced during aerobic respiration or by oxidoreductase enzymes.

These highly reactive molecules can cause damage to DNA, membranes, and other biomolecules, contributing to aging and the development of various diseases, including cancer [1,2]. H_2_O_2_ is a reactive oxidant commonly found in healthy human cells. It dismutates into water and molecular oxygen, and its overproduction and lack of regulation can cause damage to the cells and are associated with cancer. The ^·^OH radical is the most dangerous among the ROS chemicals, as it is able to reduce disulfide bonds in proteins, causing their unfolding.

On the contrary, ROS play important roles in cell survival and redox communication [3], and some ROS have been identified as potential solutions to inhibit cancer development due to their redox-communication capacities [4,5,6]: this contradictory relation between ROS and diseases reveals that they are part of a delicate balance that can provide interesting inputs for innovative therapeutic pathways.

In the cellular environment, ROS are naturally restricted by enzymes such as superoxide dismutases (SOD), superoxide reductases, and catalases [7]. These enzymes base their catalytic activity on different metals, such as iron, manganese, copper, or nickel, coordinated in their active site [8,9].

Synthetic molecules mimicking natural enzymes (“synzymes” [10]) have been made in previous studies in the field of ROS consumption.

SOD mimics based on manganese have been synthesized and evaluated in vitro and in vivo as anti-inflammatory and preventing oxidative cell damage (Failli et al. [11], Martins et al. [12], Sandhu et al. [13]), or conjugated to other cytotoxic moieties in order to enhance the efficacy since cancer cells lack enzymatic defenses against H_2_O_2_ (Prieux-Klotz et al. [14], Squarcina et al. [15]).

Catalase mimics based on manganese have been tested by Palopoli et al. [16] and Singh et al. [17], similar to catalase active sites, both in terms of dinuclear active site structure and kinetic functioning. They have obtained the kinetic constants of their compounds by cleverly merging electronic paramagnetic resonance spectroscopy (EPR) and oximetry measurements. Metal–organic frameworks with this activity have recently been developed and characterized [18].

Lessa et al. [19] have innovatively used time-resolved ESI-MS to detect intermediates of hydrogen peroxide disproportionation and to understand the catalyst self-inhibition. On this basis, in this work, we verified the consumption of the compounds when too concentrated.

Already in 1993, Baudry et al. [20] introduced Mn(III)-salen-type complexes as SOD mimics. Their work paved the way for salen-type chelators for these applications, even if it was limited to spectrophotometric and complexometric tests on a wide range of compounds. From 2019 to 2023, several new salen-type manganese complexes have been synthesized for ROS consumption [16,17,18,19]. Specifically, Richezzi et al. [21], Rayati et al. [22], and Palopoli et al. [23] have highlighted how to employ electronic spectroscopy and voltammetry to evaluate the properties of the compounds from different perspectives, as Daier et al. [24] and Segat et al. [25] did with different categories of chelators. Solomon et al. [26] deeply analyzed the redox properties and UV–visible pattern of their manganese salen-type complexes. Their work highlighted the influence of the bridge between the nitrogen atoms on the redox properties, and they even reported how to distinguish whether the complex activity was due to the metal center or to the ligand part.

In the present work, we synthesized a new salen-type aliphatic chelator, **L1**, and a known aromatic salen-type chelator, **L2**, and their respective Mn(III) complexes **C1** and **C2**. Moreover, we tested the efficacy against various ROS, including O_2_^·−^ and H_2_O_2_, and performed a preliminary kinetic study to know whether they behaved as catalysts or reacted stoichiometrically with hydrogen peroxide in specific. As Solomon et al. [26], we wanted to highlight the influence of the bridging part of the chelator and understand if the redox mechanism involved the metal center or the chelator, but related to ROS consumption and cytotoxicity effect as well, applying some of the spectrophotometric measurements reported by the mentioned works.

Many of the previous works were focused on performing purely chemical and physical characterization [16,17,18,19,20,25], while others highlighted the potential biological applications linked to ROS consumption [11,23,25,27], generally limited to contrast oxidative stress-related diseases. With our present work, we found that this kind of activity seems to correlate with cytotoxicity against cancer cells, which is not usually related to ROS consumption.

Generally speaking, we report the synthesis and characterization of Mn(III) synzymes based on different chelators, which influence the configuration of the coordination compounds and determine different efficacy towards different ROS. Additionally, these synzymes were found to be cytotoxic to lung cancer cells, and the mechanism behind this action is tentatively defined and deepened.

## 2. Results

### 2.1. Synthesis and Characterization

#### 2.1.1. Synthesis and Characterization of the Ligands

**Figure 1:**
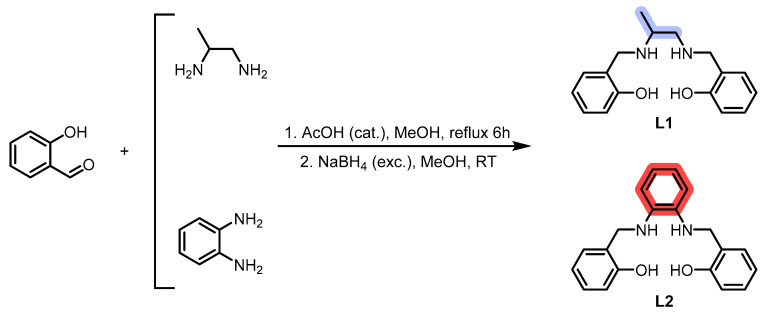
Synthesis of the chelators.

**L1** and **L2** were synthesized through imine condensation of two different diamines and salicylaldehyde, followed by reduction with an excess of sodium borohydride (NaBH_4_) in a one-pot process (see Figure 1, Section 3 and Supporting Information).

**L1** and **L2** were characterized using elemental analysis (EA), nuclear magnetic resonance (^1^H NMR, ^13^C NMR) spectroscopy, infrared (IR) spectroscopy, electrospray ionization mass spectrometry (ESI-MS), fluorescence analysis; and, for **L1**, single crystal X-ray diffraction (XRD) (see Supporting Information).

#### 2.1.2. Synthesis and Characterization of the Complexes

**Figure 2:**
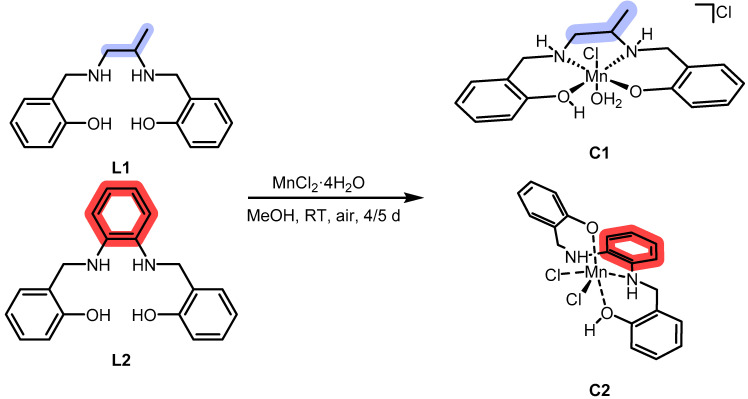
Synthesis of the Mn(III) complexes.

**C1** and **C2** were obtained by adding MnCl_2_ to methanol solutions of the chelators **L1** and **L2**, respectively. The reactions were left stirring for a few days while allowing air contact with the solution to oxidize Mn(II) to Mn(III) (see Figure 2, Section 3 and Supporting Information).

**C1** and **C2** were characterized using elemental analysis (EA), infrared (IR) spectroscopy, electrospray ionization mass spectrometry (ESI-MS), fluorescence analysis, thermogravimetric analysis (TGA) [22], electronic and fluorescence spectroscopy, paramagnetic NMR spectroscopy [21,28], and cyclic voltammetry (CV). We assume that the phenolic proton is in a tautomeric equilibrium involving both the phenolic oxygen atoms since their chemical arounds are identical in both molecules. Fluorescence spectroscopy characterization was necessary to ensure no interference in the biological assays (see Section 2.3 and Section 3).

**Figure 3:**
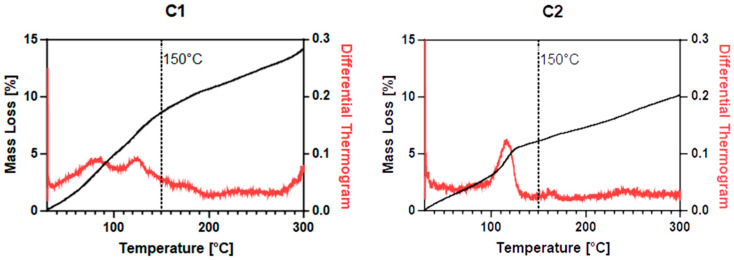
Thermogravimetric analysis of **C1** (on the left side) and **C2** (on the right side). Solid compounds were put in a platinum crucible and heated up to 300 °C, after which decomposition appeared to occur.

Thermogravimetric analysis (see Figure 3) is a useful tool to assess the nature of water molecules in a complex: in fact, one can distinguish between crystallization and coordination molecules by detecting the temperature at which they are released. For this discrimination, 150 °C is considered to be the threshold temperature for crystallization water, whereas, above 150 °C, coordination water molecules are to be released. In our case, both the complexes visibly decompose at 300 °C. Therefore, we chose it as the maximum temperature. In the case of **C1**, we have an 8.7% mass loss at T < 150 °C, corresponding to three molecules of crystallization water, a 2.2% mass loss between 150 °C and 200 °C, corresponding to a coordinated water molecule, and a 3.5% afterward, corresponding to an HCl molecule released by the complex. In the case of **C2**, we have a 6.1% mass loss at T < 150 °C, corresponding to two molecules of crystallization water, and a 4.1% afterward, corresponding to an HCl molecule released by the complex.

### 2.2. Chemical and Physical Assays

#### 2.2.1. Stability Assays

The stability of the complexes (**C1** and **C2**) and ligands (**L1** and **L2**) in a medium simulating in vivo conditions was tested. They were dissolved in phosphate buffer (PBS) with 1% dimethyl sulfoxide (DMSO) at 10^−4^/10^−5^ mol/L and incubated at 37 °C for 24 h. These solutions were monitored using electronic spectroscopy.

**Figure 4:**
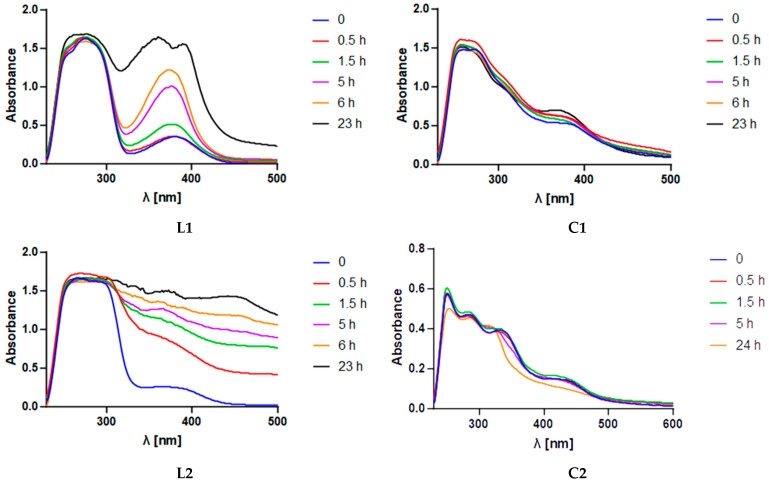
UV–visible spectra (PBS and 1% DMSO, 10^−4^/10^−5^ mol/L) used to test the stability of the complexes and the related ligands in a 24 h period. The compounds were incubated at 37 °C at atmospheric pressure.

As shown in Figure 4, the coordination compounds (**C1** and **C2**) appeared to undergo minimal changes over the 24 h period, whereas the ligands (**L1** and **L2**) showed signs of deterioration. These interesting results suggest that the complexes are stable, indicating that the metal center is needed to stabilize both of the chelators.

#### 2.2.2. Complexes Structural Information and Computational Studies

The structural features of **C1** and **C2** were investigated using various techniques, including electronic and NMR spectroscopy, EA, TGA, and CV.

Despite many attempts in very different solvent systems and at different temperatures, only amorphous precipitates were formed, therefore not allowing an XRD characterization of the complexes.

The electronic spectra of **C1** and **C2** in DMSO display the same UV–visible pattern reported in the literature [17,21,27,29]: π-π* intra-ligand transitions around 280/290 nm (**C1**: 276 and 309 nm; **C2**: 278 nm and 335 nm), a less intense band at approximately 400 nm (**C1**: 390 nm; **C2**: 436 nm), which can be related to ligand-to-metal charge transfer (LMCT) transition from the p_π_ orbitals of the phenolate to the partially filled d_π*_ orbitals of Mn(III), and partially hidden Laporte forbidden *d-d* transitions between 400 and 650 nm.

For both complexes, we performed a tentative deconvolution using Fityk (see Supporting Information).

Considering that Mn(III) octahedral coordination forms a D_4h_ octahedron, influenced by Jahn–Teller compression along the *z*-axis, the spin-allowed transitions observable with monodentate or symmetrical ligands are typically ^5^E_g_←^5^B_1g_, ^5^B_2g_←^5^B_1g,_ and ^5^A_1g_←^5^B_1g_. In the literature [21,30], they are reported in decreasing order of energy, and in addition, ^5^B_2g_←^5^B_1g_ is related to the crystal field splitting energy, which helps compare the stabilization of the complexes.

In the case of **C1** and **C2**, the lack of a symmetry center reduces the symmetry grade and therefore they must be attributed to *C_1_* and *C_S_* symmetry groups, respectively. Deconvolution allowed us to identify two out of three transitions for both compounds, with the first likely hidden by the LMCT absorption band: **C1** at 449 (ε [cm^−1^M^−1^] = 313) and 493 nm (ε [cm^−1^M^−1^] = 150) and **C2** at 522 (ε [cm^−1^M^−1^] = 577) and 566 (ε [cm^−1^M^−1^] = 247) nm.

It is noteworthy that the second transitions (**C1** at 449 nm and **C2** at 522 nm) are related to the crystal field splitting in the literature [21,30] and indicate a significant difference in stabilization between the complexes. This suggests that in **C1** the ligand is in a *trans* configuration, while in **C2** the ligand is in a *cis* configuration, making the latter more constrained and therefore more destabilized.

NMR spectroscopy can be useful for investigating ligand configuration around a paramagnetic metal such as Mn(III) [21,31,32]. This cation is paramagnetic due to its two unpaired electrons, causing some protons of the chelator to experience a strong paramagnetic effect that significantly shortens the relaxation time [31]. The spectral pattern for protons of the phenol arms might result from σ/π-spin delocalization through the C bonds of the coordinated phenolate [32].

**Figure 5:**
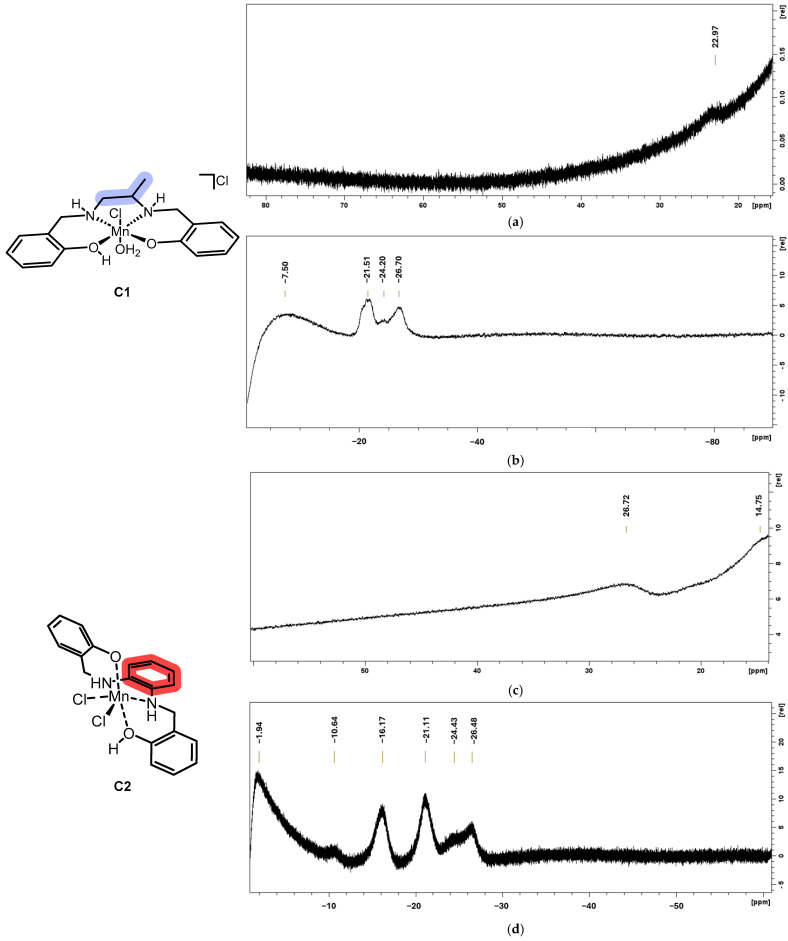
NMR spectra (Bruker sequence: zg30) of **C1** ((**a**) from 30 to 0 ppm, SW = 200 ppm, O1P = 6.35 ppm, NS 100; peak at 23 ppm; (**b**) from 0 to −30 ppm, SW = 100 ppm, O1P = −50 ppm, NS = 150; peaks at −7.5, −22, −24, and −27 ppm) and **C2** ((**c**) from 13 to 32 ppm, SW = 60 ppm, O1P = 41 ppm, NS 100; peaks at 27 and 15 ppm; (**d**) from 0 to 30 ppm, SW = 60 ppm, O1P = −31 ppm, NS = 150; peaks at −1.9, −11, −16, −22, −24, and −26 ppm). The spectral range of routine ^1^H-NMR was excluded for clarity.

If the chelator is in a *trans* or *cis-α* conformation, the protons of the two phenolic groups are symmetrical, resulting in a simple pattern. On the other hand, if the system is in a *cis-β* conformation, the protons of the two different aromatic rings are non-equivalent, causing a larger number of signals [21]. The NMR spectra of **C1** show a simple pattern (see Figure 5) with two intense signals (−21.51, −26.70 ppm) and some broader signals (−7.50, 22.97, and −24.20 ppm). This suggests that the phenolate groups are symmetrical, and the additional broad signals can be related to the phenolic hydrogen and/or the aliphatic bridge nuclei. In contrast, the NMR spectra of **C2** display a doubled pattern: four intense signals (−1.94, −16.17, −21.11, and −26.48 ppm) and 4 broad/intermediate peaks (26.72, 14.75, −10.64, −24.43 ppm).

**C1** might be either *trans* or *cis*-*α*, while **C2** would be *cis*-*β*.

Combining these observations with the electronic spectra interpretation and the CV analysis (see next), it seems that the most plausible conformation for **C1** is *trans*.

Voltammetric characterization is a crucial step in rationalizing the redox synzyme and can provide insights into structural features. To this end, the complexes were characterized by cyclic voltammetry (CV) in a dichloromethane (DCM)/dimethylformamide (DMF) 9:1 mixture to enhance their solubility and improve the resolution of the signals.

**Figure 6:**
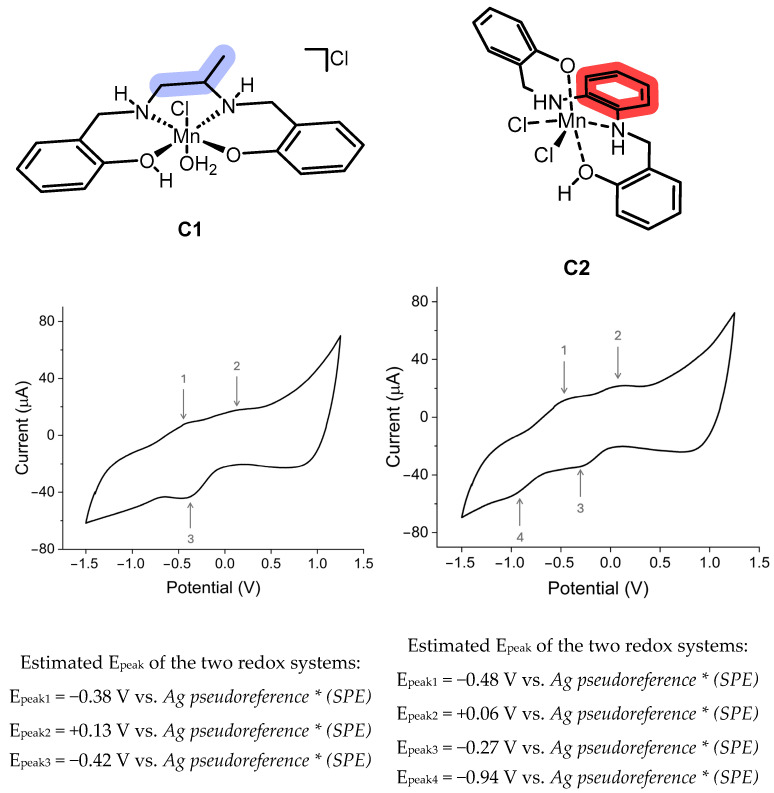
Cyclic voltammograms of 1 mmol/L complexes in DCM:DMF 9:1 with 0.1 mol/L tetrabuthylammonium hexafluorophosphate (Bu_4_NPF_6_) as the supporting electrolyte were recorded at a scan rate of 20 mV/s and a temperature of 20 °C. * The voltammograms were acquired using screen-printed electrodes (SPE) with a carbon working and counter electrode and a silver pseudoreference electrode. Ferrocene was used as an internal standard and exhibited an E_1/2_ of +0.5 V with respect to the silver pseudoreference electrode.

As shown in Figure 6, the voltammograms indicate the coexistence of two redox systems, possibly related to the Mn(III)/Mn(II) and Mn(IV)/Mn(III) systems. The literature data [21] indicate that redox potentials are lower for a *cis-β* configuration in comparison to a *trans* configuration. Based on these considerations, the voltametric pattern of **C1** and **C2** suggests *trans* and *cis-β* configurations, respectively.

The attempt to study also by means of electronic paramagnetic resonance spectroscopy (EPR) the interactions of the newly synthesized molecules with radical species revealed the presence of Mn(II) signals. On the other hand, cyclic voltammetry demonstrated a predominant majority of Mn(III) in place of trace amounts of Mn(II). The first voltammetric scans of both **C1** and **C2** show an intense cathodic current, indicating the reduction of the metal center. In addition, the anodic peak was not noticed when the starting potential of the first CV scan was fixed at the equilibrium potential (open circuit), thus revealing that the compounds are already oxidized at the beginning (see Table S1). Based on those findings, we can assume that our Mn(III) complexes were contaminated by Mn(II) impurities detected by EPR.

Computational studies allowed us to have a deeper understanding of the characteristics of complexes **C1** and **C2**. These studies were performed in light of the results of the previously reported analyses, which indicated the most plausible structure for both the compounds **C1** and **C2**.

Computational studies focused on obtaining maps of electrostatic potential (MEP) and images of the highest occupied orbitals (HOMO) and of the lowest occupied orbitals (LUMO).

**Figure 7:**
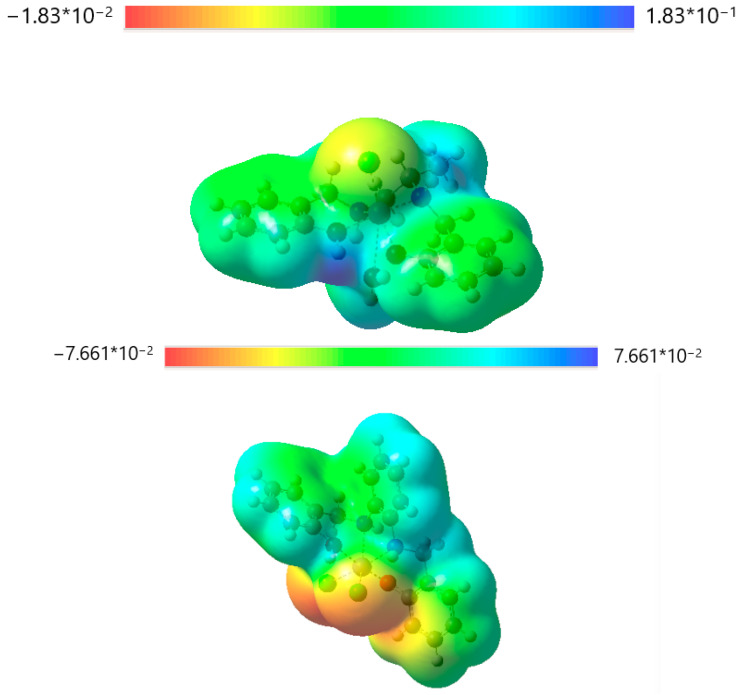
**C1**, above, displays a homogeneous distribution of its net positive charge. **C2**, below, shows a much more variegated pattern, with a high negative charge density on the chloride atoms and a positive charge density on the hydrogen atoms, primarily of the phenyl groups.

MEPs (reported in Figure 7) show that the **C1** positive charge (reported in light blue/blue color) is homogenously distributed all over the molecule, apart from a slight electron density on the chloride surface (in yellow color). From a biological point of view, this can be interesting to enhance the permeability of the cell barriers. **C2** is neutral, so its permeability is already facilitated. On the other hand, **C2** displays a heterogeneous surface, with the partial negative charge (in orange/red color) concentrated in the chlorides area and the partial positive charge (in light blue color) in the hydrogen atoms of the phenyl groups.

**Figure 8:**
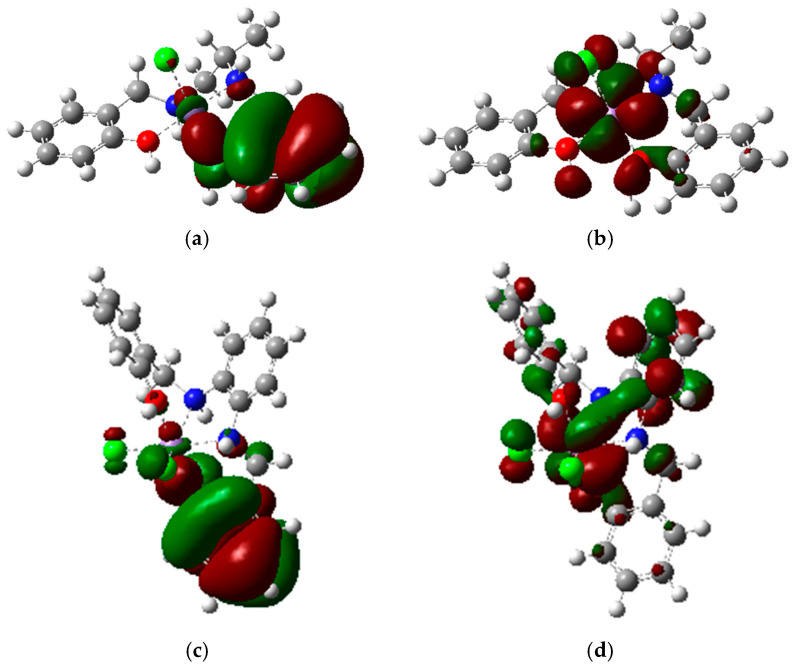
(**a**) HOMO of **C1**; (**b**) LUMO of **C1**; (**c**) HOMO of **C2**; (**d**) LUMO of **C2**. These calculations seem to indicate that HOMO is mainly localized on one of the phenolate groups, whereas **C1** LUMO involves uniquely the metallic center and its surroundings, and **C2** LUMO involves the metallic center and the diaminic part of the chelator.

Calculated HOMO and LUMO (see Figure 8) can provide a superficial indication of the expectation regarding one-electron exchanges, such as those that can occur with interaction with ROS. The electrons released through oxidation are described by the HOMO orbital, whereas the LUMO orbitals describe the electrons acquired through a reduction process. The HOMO of both **C1** and **C2** are mainly located in the phenolic group, specifically the deprotonated one in the context of the tautomeric equilibrium. This indicates that **C1** and **C2**’s first oxidation might primarily involve the chelator and only secondarily the metallic center.

The LUMO of **C1** involves both the metallic center and the coordinating parts of the chelator, indicating that the reduction of **C1** might predominantly occur on the manganese atom and in its immediate surroundings.

The LUMO of **C2** is spread between the chloride atoms and the diaminic aromatic bridge passing through the metal center; therefore, the reduction of this complex is expected to involve both the ligand and the manganese atom.

#### 2.2.3. Anti-ROS Activity Monitored by Electronic Spectroscopy [21,25], Clark-Type Electrode Oximetry [16], and ESI-MS [19]

UV–visible spectroscopy and oximetry are useful for testing the capacity of newly synthesized compounds to consume radicals and oxidants. **C1** and **C2** were evaluated against species differing in size and reactivity, namely (from largest to smallest) 2,2-diphenyl-1-picrylhydrazyl (DPPH), H_2_O_2_, O_2_^−·^, and ^·^OH, using electronic spectroscopy assays. A Clark-type oximeter was used to monitor oxygen development during H_2_O_2_ dismutation, providing insights into the ongoing mechanism, and an ESI-MS time-dependent experiment was performed in addition.

2,2-Diphenyl-1-picrylhydrazyl (DPPH) is a large and stable radical compound. Its consumption can be detected via a colorimetric method because it is intensely purple in color, while its reduced form is light yellow. DPPH neutralization is monitored by observing the absorption band at 517 nm. A decrease in this absorption indicates quenching of the radical. This test is useful for verifying the capacity of a compound to consume bulky radicals in general. The absorbance values of a DPPH solution and mixtures at different ratios of this radical and the target compound are reported as radical scavenging activity (RSA; see Equation (1), Section 3.8.1). Previous studies have employed this assay to test the antioxidative properties of Schiff base complexes of various transition metals, including Mn(III) [25,32,33].

**C1** and **C2** demonstrated very different activities towards this radical. **C1** was substantially inactive, whereas **C2** showed effectiveness proportional to its concentration (see Table 1 and Supporting Information). The ligands were also tested to determine if they influenced these results: **L1** showed no efficacy, like **C1**, whereas **L2** demonstrated even higher efficacy than its complex.

**C2** was found to have a comparable reactivity with the [Mn(salen)Cl] reference [21]. Due to **C2**’s high stability (see Section 2.2.1), it is unlikely that its efficacy is caused by a fraction of the ligand being released into the solution. Therefore, we hypothesize that the reaction mechanism involves either the metal center (as in natural enzymes) or the exposed part of the aromatic ligand. On the other hand, the aliphatic bridge seems to impede activity towards large radicals like DPPH, as neither **C1** nor its chelator displayed relevant activity. It must be noted that **L2** is a way better scavenger than all the other compounds: the main obstacle to further studies on this compound is its instability (see Figure 3) even after limited time intervals, making it difficult to be employed as a potential drug.

**Figure 9:**
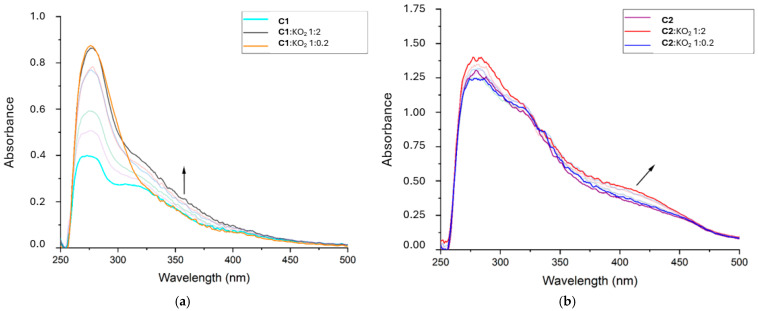
Electronic spectra of **C1** (**a**) and **C2** (**b**) mixed at different ratios with O_2_^-·^ anion revealing the same hyperchromic effect on both π-π* and LMCT bands but with evidently different intensities. Stock solutions (330 μmol/L) of the complexes and KO_2_ (660 μmol/L) were prepared in dry DMSO. Superoxide concentration was evaluated by Lambert–Beer Law (ε = 2686 M^−1^ cm^−1^ at λ = 280 nm). UV–visible spectra of the solutions (600 μL), containing different complex-to-superoxide anion ratios: 1:2, 1:1.6, 1:1.2, 1:0.8, 1:0.4, 1:0.2.

The interaction of **C1** and **C2** with O_2_^−·^ was monitored via electronic spectroscopy at different concentrations (see Section 3). The superoxide anion is smaller and more reactive than DPPH. In this test, superoxide was mixed with the target compounds at different ratios, and by comparing subsequent spectra, it was possible to detect potential changes due to the interaction (see Figure 9).

As previously reported [24,25], if superoxide interacts with the metal center, it is expected to have a Mn(III) reduction to Mn(II). This causes the disappearance of the LMCT band at 300–400 nm. On the other hand, when the charge is delocalized on the ligand [23], both π-π* and LMCT are expected to increase.

It appears that in the complexes we studied, the main interaction involves both metal and ligand. Even if we did not witness LMCT band elimination, some modifications occurred at this level as well: this would indicate that the metal center was not exclusively reduced, but the ligand reduction influenced the coordination.

**C1** exhibited a more notable hyperchromic effect at both the π-π* and LMCT bands than **C2**.

This result appears to be in contrast with the literature [26], in which aromatic scaffolds have better stabilized the radical and therefore have expressed a more intense increase in absorbance.

It is possible to hypothesize that the differences in structures influence the different behaviors. In fact, it can be assumed that **C1** delocalizes better radicals due to its *trans* configuration. Based on the coplanarity of the two phenolic groups and on the connection through the oxygen atoms and the Mn center, **C1** offers a molecule-wide system to stabilize the unpaired electron. On the other hand, **C2** seems to be in a *cis* configuration, which does not allow delocalization larger than a single aromatic ring. In addition, being **C1** a positively charged compound surely has had an impact on its effectiveness compared to the neutral **C2**.

Computational studies (see Section 2.2.2) indicated that the LUMO of both complexes involved both the metal center and the chelator, mainly the functional groups coordinating the manganese atom. This experiment showed a certain coherence with those results since the reduction impacted the whole absorption spectrum.

Our complexes’ capacity to be reduced by ·OH was tested via electronic spectroscopy. ^·^OH was produced in situ through the Fenton reaction (see Section 3) by mixing iron(II) sulfate and hydrogen peroxide. UV–visible spectra were taken every 12 s for 5 min, and once again it seems that **C1** and **C2** behave differently.

**Figure 10:**
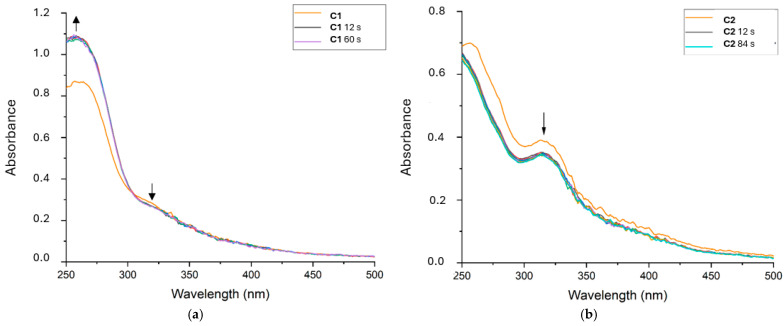
UV–visible spectra of **C1** (**a**) and **C2** (**b**) in the presence of ·OH recorded every 12 s for 5 min. The reactivity of **C1** and **C2** with ^·^OH was moderate but very rapid for both. **C1** exhibited a hyperchromic deviation at the π-π* absorption and a slight hypochromic effect at the LMCT absorption, whereas **C2** showed a hypochromic effect at both wavelengths. A stock solution was prepared for each complex (**C1**, **C2**) in PBS buffer (150 μmol/L, 1% DMSO). 150 μL of a 1 mM solution of FeSO4 and 50 μL of a 10 mM solution of H_2_O_2_ were diluted with 500 μL of distilled water into two distinct solutions. 100 μL of such solutions were added to 500 μL of complex stock solution.

Both compounds exhibited moderate yet rapid activity towards hydroxyl radicals, but with important differences: as shown in Figure 10, **C1** exhibited a hyperchromic variation at the π-π* absorption band and a slight hypochromic effect at the LMCT absorption band, whereas **C2** underwent a very slight hypochromic effect at both wavelengths.

**C1** evident increase in absorption at low wavelengths and no increase of the LMCT band may be correlated to direct oxidation of the ligand. This behavior would coincide with the computational results, which report that the HOMO is mainly localized on the ligand (see Section 2.2.2).

**C2**, instead, underwent slight changes all along the absorption spectrum. This uniform change can be related either to an interaction involving both Mn(III) and the ligand **L2** with hydroxyl radical, or simply to no reaction at all and a slight precipitation event.

**C1** and **C2** were mixed in a solution with H_2_O_2,_ and UV–visible spectra were taken every 12 s for 5 min, followed by an additional recording after 30 min.

**Figure 11:**
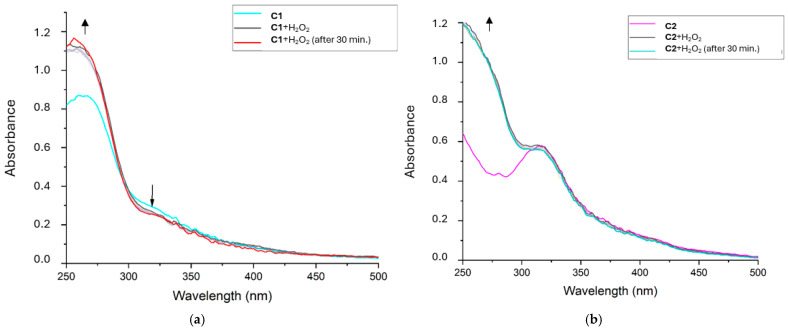
UV–visible spectra of **C1** (**a**) and **C2** (**b**) before and after the addition of hydrogen peroxide revealed almost no consumption of the complexes examined. Stock solutions at 150 μmol/L of complexes (**C1**, **C2**) were prepared in PBS (with 1% DMSO), while H_2_O_2_ was diluted in distilled water to obtain a 150 mmol/L stock solution. 500 μL of complex solution and 100 μL of solvent or H_2_O_2_ stock solution were mixed, and spectra were registered in the 250–750 nm window every 12 s for 5 min, plus a final spectrum at 30 min to check possible modifications.

These spectra reveal that the compounds do not change in correspondence to the LMCT absorption band. On the other hand, the absorbance increased in correspondence to the π-π* absorption (see Figure 11). It seems that some kind of degradation occurred at these conditions (125 μmol/L of compound and 25 mmol/L of hydrogen peroxide). Computational studies (see Section 2.2.2) indicated that the HOMOs were mainly located on the ligands, and witnessing that the main change due to oxidation actually happened at the ligand level can be an indication of the mechanism of the reaction.

The Clark-type oximeter was used for detecting the oxygen produced via hydrogen peroxide dismutation over a period. This provides information about the existence of a turnover of the process and helps analyze the kinetics as well, as reported by Palopoli et al. in 2000 [16]. We employed an extremely simple setup without solvent degassing or previous saturation. The hydrogen peroxide concentration was kept constant and in vast excess in all measurements, while the concentration of **C1** and **C2** varied from 15 to 300 μmol/L (see Section 3 and Supporting Information). This experiment was targeted to reckon a catalytic activity and to preview deeper analysis of the catalytic constants in the future.

It appears that both **C1** and **C2** have catalytic activity since any quantity of complexes caused the formation of a higher amount of oxygen molecules compared to the complex stoichiometric concentration (see Table S2).

However, at the highest concentration used in our experiments, it seems that the activity of these catalysts is reduced. This catalyst self-inhibition is known in the literature for other Mn-based synzymes [16,24].

**Figure 12:**
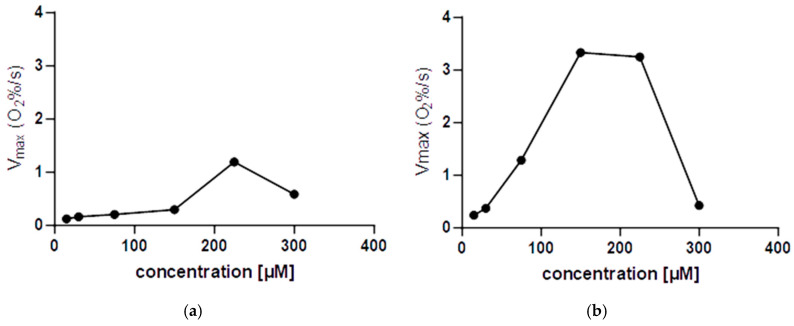
Oximetry results. V_max_ at different compound concentrations ((**a**) **C1**; (**b**) **C2**) suggests a possible poisoning of the catalyst by the same oxygen produced during hydrogen peroxide dismutation. Here, V_max_ refers to the slope at t_0_ in a graph of O_2_ [%] vs. time [s], as described in Materials and Methods. Solutions at 1% DMSO in PBS, H_2_O_2_ 107 mmol/L.

This self-inhibition occurred at higher concentrations of both hydrogen peroxide and complex, compared to the UV–visible test: 107 mmol/L vs. 25 mmol/L for H_2_O_2_ and approximately between 200 and 300 μmol/L for both complexes with Clark-type oximeter and 125 μmol/L in the UV test (see Figure 12).

It could be related to Mn(IV)-oxo byproducts resulting from the high local concentration of oxygen produced in situ or to Mn(III)(*μ*-O)_2_Mn(IV) dimers formed by both oxygen derivatives and Mn synzyme itself. This latter option is excluded because UV tests never showed the 500 nm absorption band related to Mn(III)(*μ*-O)_2_Mn(IV) byproduct [28,32].

In addition, higher H_2_O_2_ concentration seems to push complexes to catalyze hydrogen peroxide disproportionation in place of being consumed by molecular oxygen.

To verify the consumption of **C1** and **C2** in these conditions, we performed a time-dependent ESI-MS study (Lessa et al. [19]). This method allows for the recognition of the depletion of molecular species and possibly for the identification of the products of this deterioration.

In our case, we employed a low-resolution MS to obtain fast feedback about this kinetic problem. We prepared the solutions at the same concentrations at which the oximeter revealed a deviation from growth, and three measures were taken subsequently at 3 min intervals.

**Figure 13:**
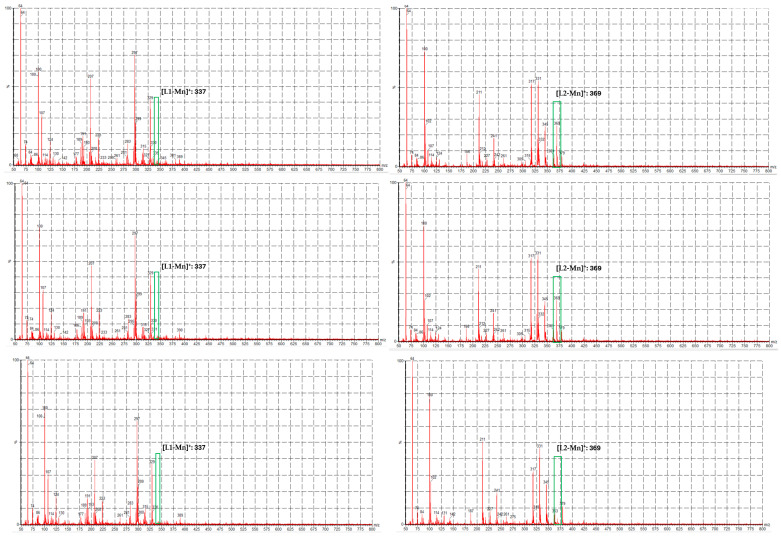
ESI-MS spectra of **C1** (**left**) and **C2** (**right**) dissolved in CHCl_3_/MeOH 1:1 at 300 μmol/L treated with H_2_O_2_ 0.107 mol/L at 0, 3, and 6 min. On the left, the molecular ion of **C1** is absent, and on the right, the molecular ion of **C2** decreases in intensity with time.

The molecular ion of **C1** is absent in all the spectra (see Figure 13), whereas the molecular ion of **C2** decreases in intensity. Although it was not possible to identify byproducts, it seems that hydrogen peroxide mixed with concentrated **L1** and **L2** degrades the same complexes.

These studies suggest that **C1** and **C2** catalyze the dismutation of H_2_O_2_ in a concentration-dependent manner. It is noteworthy that up to a certain limit, their efficacy decreases, possibly due to poisoning by molecular oxygen.

#### 2.2.4. Determination of Proton Dissociation Constants

Proton dissociation constants of **L1** were determined in aqueous solution using glass–electrode potentiometry. The pKa values resulted in 5.78(1), 8.25(1), 9.64(1), and 10.43(1). The first two values can be associated with the deprotonation of the secondary amine groups, while the other two are associated with the phenolic OH groups. It should be noted, however, that these pKa are close in their values, and therefore the deprotonation processes are partially overlapped in solution. A representation of the distribution diagram is reported in Figure S19 of Supporting Information.

We attempted the determination of the pKa values for **L2**, but we observed an instability in the registered electromotive force during the titration in the form of spikes in the titration curve (at pH ca. 3.5). We have attributed this behavior to the air sensitivity of the ligand at these pH values.

It is noteworthy that the ligands behave as very weak acids (see. pKa values). However, the Mn(III) products **C1** and **C2** can be isolated without the addition of a base to neutralize the ligand. This behavior is an effect of the predicted high stability of the metal complexes, likely due to the presence of hard and intermediate donor atoms and to the preorganization of the ligand to form three chelate rings.

### 2.3. Biological Evaluation

#### 2.3.1. Cytotoxicity Assays and Other Cellular Analyses

The growth and viability of A549 cells treated with ligands and complexes were evaluated using the MTT assay. Additionally, cell count analysis was performed using Trypan blue to exclude non-viable cells after 24 and 48 h of compound exposure. Treatments with **L1**, **C1**, **L2**, and **C2** significantly inhibited cell growth and affected cell viability in a concentration-dependent manner, except for **L1** and **C2** treatment at 24 h. All treatments, however, resulted in inhibition of cell growth after 48 h. The IC_50_ values related to the ligands and complexes are shown in Table 2.

The cytofluorimetric analysis conducted after exposing the cells to the compounds at the IC_50_ concentration highlighted a significant decrease in the G_2_M phase starting from 12 h for **L1**, **C1,** and **C2**, while the percentage of cells treated with **L2** was similar to that of the control. The other phases of the cell cycle also experienced a significant decrease, especially after treatment with **C2** (see Table 3).

**Figure 14:**
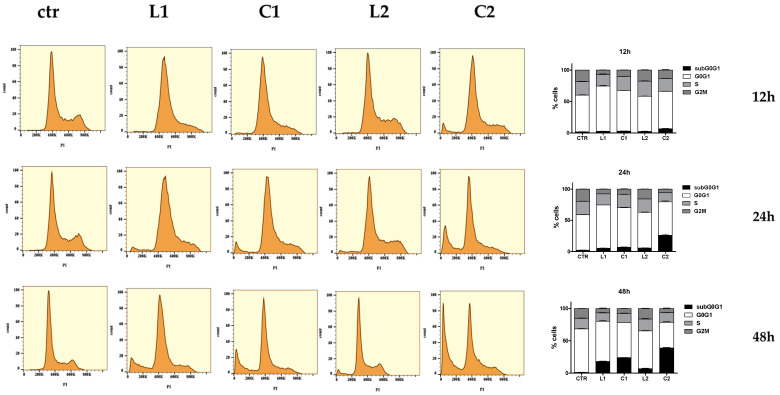
Monoparametric DNA analysis of the cellular cycle distribution after 12–48 h treatment with compounds. Three distinct phases could be recognized in the proliferating cell population, corresponding to different peaks: G_0_/G_1_, S, and G_2_/M phase.

**Figure 15:**
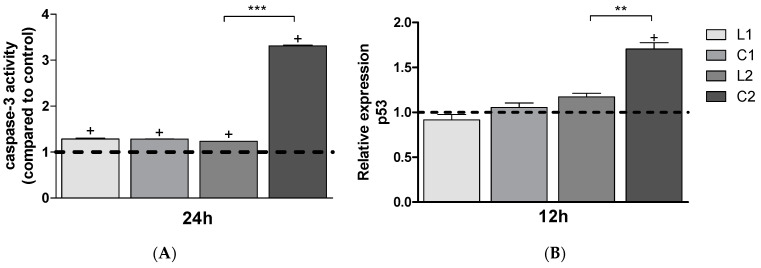
Effects of compounds on caspase-3 activity (**panel A**) and p53 relative expression (**panel B**) after 24 h or 12 h treatment. Values are mean ± SD of three separate experiments, each carried out in triplicate (dashed line = CTR). Values vs. control = sample value/control value. Statistical significance is indicated as follows: significantly different from untreated control ^+^ *p* < 0.001, and C2 vs. L2 ** *p* < 0.01; *** *p* < 0.001.

Of particular importance in the analysis of cell cycle progression is the presence of a subG_0_G_1_ peak starting from 12 h for the **C2** complex, which reaches a percentage of 38% at 48 h. The other compounds also induce apoptosis, although to a lesser extent (Figure 14 and Table 2). These data were confirmed by the caspase-3 activity analysis, which indicated the presence of apoptosis in response to the treatments. In agreement with these data, the evaluation of the expression of the p53 gene was significantly upregulated by all treatments (Figure 15).

**Figure 16:**
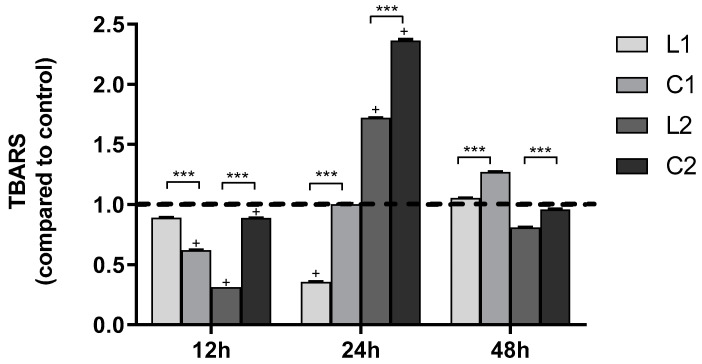
TBARS in A549 cells after 12–48 h treatment of compounds. Values are mean ± SD of three separate experiments, each carried out in triplicate (dashed line = CTR). Values vs. control = sample value/control value. Statistical significance is indicated as follows: significantly different from untreated control **^+^** *p* < 0.001, and **C** vs. **L** *** *p* < 0.001.

Oxidative stress was assessed using the TBARS (Thiobarbituric Acid Reactive Substances) assay, commonly employed to measure lipid peroxidation levels. In A549 cells, TBARS was evaluated after treatment with ligands and complexes at 12, 24, and 48 h. A significant increase in lipid peroxidation of the cell membrane levels was observed following treatment at 24 h with **L2** and **C2** compounds and at 48 h with **L1** and **C1** compounds (Figure 16). TBARS values of **L2** and **C2** after 48 h decreased because cells recovered also thanks to the high expression of protective enzymes.

**Figure 17:**
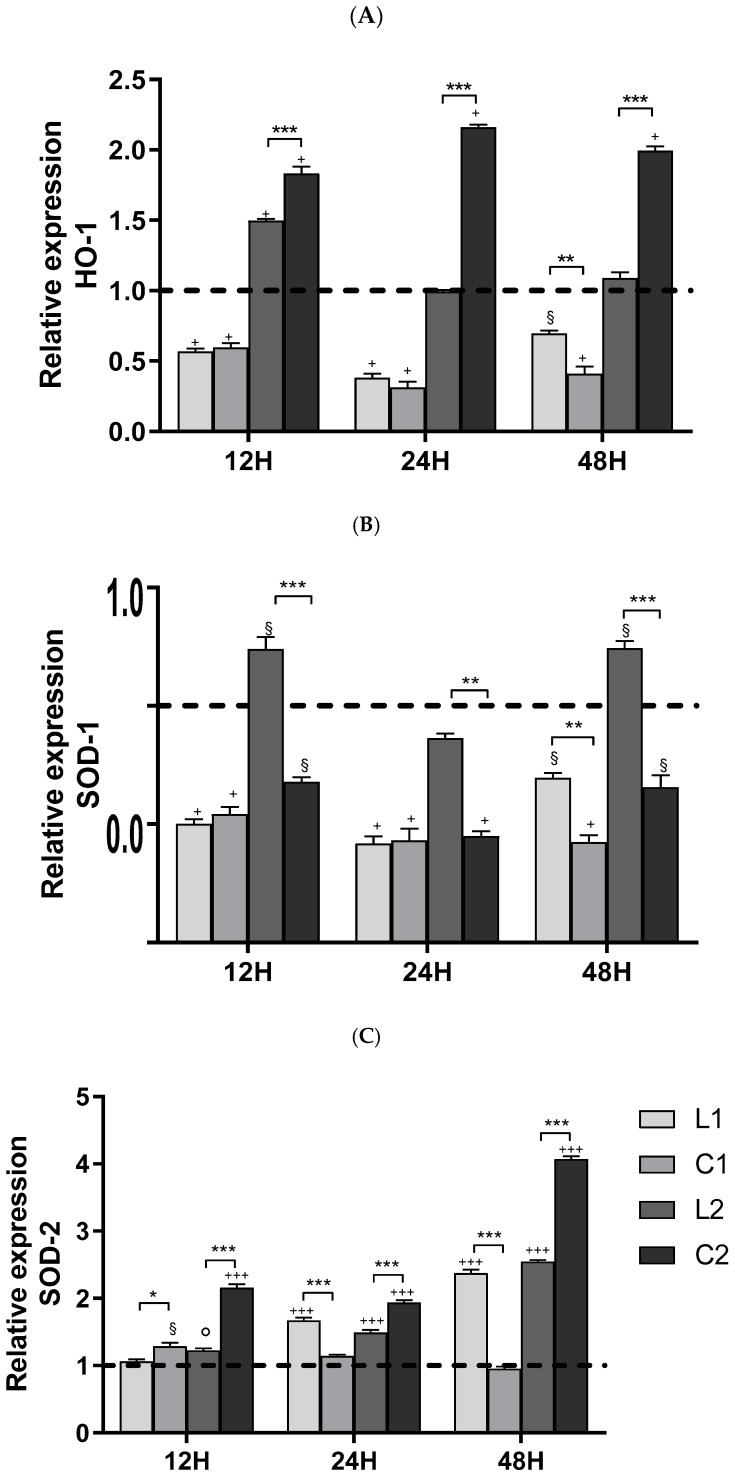
Effect of ligands and complexes on HO-1, SOD-1, and SOD-2 gene expression (panels (**A**–**C**), respectively) after 12, 24, and 48 h of treatment. Values are mean ± SD of three separate experiments, each carried out in triplicate (dashed line = CTR). Statistical significance is indicated as follows: ^+^ *p* < 0.001, ^§^ *p* < 0.01, ° *p* < 0.05, ^+++^ *p* < 0.001 values vs. control*;* * *p* < 0.05, ** *p* < 0.01, *** *p* < 0.001 **C** vs. **L**.

This increase was further supported by the upregulation of HO-1, and SOD-2 gene expressions, mainly after treatment with **L2** and **C2** compounds at 12, 24, and 48 h. SOD-1 expression was upregulated only by treatment with **L2** (Figure 17). The upregulation of these genes suggests that A549 cells respond to the increased oxidative stress by activating their antioxidant defense mechanisms. In this study, apoptosis was also evaluated by evaluating caspase-3 activity, which confirmed the presence of apoptosis in response to the treatments.

**Figure 18:**
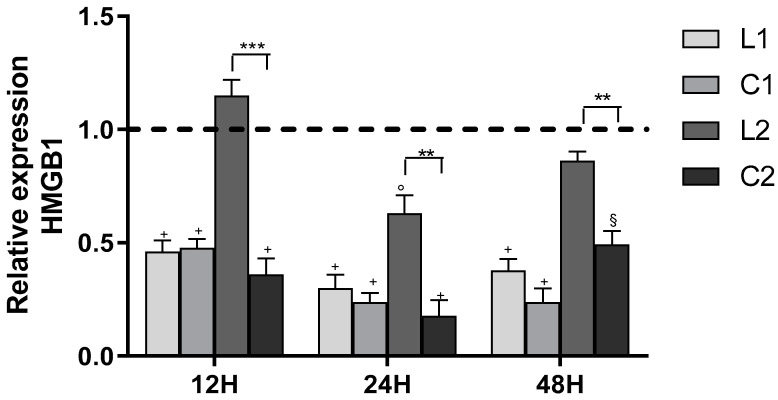
Effect of ligands and complexes on HMGB1 gene expression after 12, 24, and 48 h of treatment. Values are mean ± SD of three separate experiments, each carried out in triplicate (dashed line = CTR). Statistical significance is indicated as follows: ^+^ *p* < 0.001, ^§^ *p* < 0.01 ° *p* < 0.05 values vs. CTR; ** *p* < 0.01, *** *p* < 0.001 **C** vs. **L**.

All treatments were able to induce downregulation of the expression of the gene coding for HMGB1 (*p* < 0.001) (Figure 18). HMGB1 is a structural, non-histone chromatin protein that plays a fundamental role in chromatin remodeling and the development of the inflammatory process. The modulation of inflammatory responses occurs through the signal transduction pathway mediated by specific receptors such as RAGE and Toll-like receptors (TLR) 2 and 4. The binding of HMGB1 to these receptors leads to cell proliferation, increased angiogenesis, invasion, metastasis, the ability to avoid apoptosis, and heightened inflammation. Reducing its expression could create conditions favorable for blocking tumor proliferation.

#### 2.3.2. Toxicological Analysis with *Galleria mellonella*

**Figure 19:**
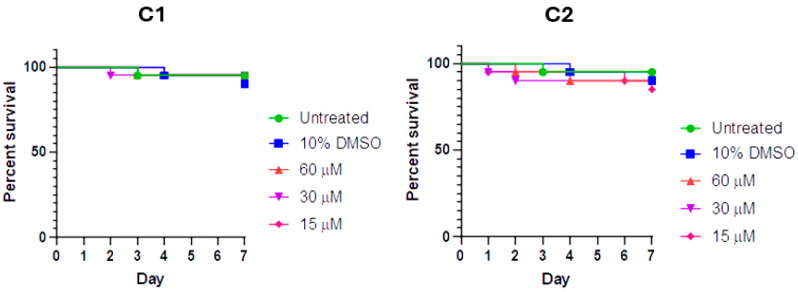
Survival of *G. mellonella* (n = 20) recorded over a 7-day period post-injection of 10 μL of concentrated **C1** and **C2** solutions, diluted in larva to the reported concentrations. Both complexes appear not to be significantly harmful in vivo.

The *Galleria mellonella larvae* test is an affordable, easy, and reliable methodology to evaluate the in vivo toxicity of newly synthesized drug candidates [34,35,36]. In this study, the larvae were injected with varying concentrations of the compounds and incubated at 37 °C for a week. The larvae were daily monitored for survival and the percent survival is reported in Figure 19.

The mortality rate of larvae injected with either **C1** or **C2** did not appear to be higher than that of non-injected larvae, suggesting that these drug candidates exhibit selectivity even in vivo (see Section 3).

## 3. Materials and Methods

### 3.1. Reagents and Instrumentation

All common laboratory chemicals were purchased from commercial sources and used without further purification: 2-hydroxybenzaldehyde, ≥98.0% (Glentham Life Science, Planegg, Germany), 1,2-diaminopropane, >99% (Merck, Darmstadt, Germany), o-phenylenediamine, 99.0% (Fluorochem, Hadfield, UK), manganese (II) chloride tetrahydrate, 99% (Carlo Erba), sodium borohydride, 99% (ThermoScientific, Waltham, MA, USA), hydrogen peroxide 30% (Merck, Darmstadt, Germany). NMR was recorded on a Bruker Anova spectrometer at 400 MHz (Billerica, MA, USA), with chemical shift reported in δ units (ppm). NMR spectra were referenced relative to residual NMR solvent peaks. The solvent used in the spectra’s acquisitions is DMSO-*d*_6_. The FT–IR measurements were recorded on Nicolet 5PC FT–IR (Rodano, MI, Italy) in the 4000–400 cm^−1^ range, equipped with the ATR accessory. Elemental analyses were performed using the Thermofisher Scientific Flashsmart CHNS Elemental Analyzer (Rodano, MI, Italy). ESI-MS were recorded on a Waters Acquity Ultraperformance ESI-MS spectrometer with a Single Quadrupole Detector (Sesto San Giovanni, MI, Italy). UV–visible spectra were collected using a Thermofisher Scientific Evolution 260 Bio Spectrophotometer (Rodano, MI, Italy), using quartz cuvettes of 1 cm path length. Clark electrode kinetic analysis was conducted with a Knick SE715 Memosens oxygen sensor (Berlin, Germany) plugged into a Knick Portavo 907 Multi multimeter (Berlin, Germany). Fluorescence spectra were collected with an FLS1000 Edinburgh Instruments fluorometer (Edinburgh Instruments Ltd., Livingston, UK) equipped with a 450 W Xenon lamp as the excitation source, using standard 1 cm × 1 cm 3 mL quartz cuvettes. To minimize inner-filter effects, samples with optical density < 0.1 were analyzed. All the emission spectra were duly corrected for the wavelength-dependent responsiveness of the detection path. Cyclic voltammetry (CV) experiments were carried out using a μStat 8000 Multi Potentiostat/Galvanostat (Metrohm Italiana s.r.l., Origgio, Italy) on screen-printed electrodes with carbon working and counter electrodes and silver pseudoreference (SPCE DRP-C110, Metrohm Italiana S.r.l., Origgio, Italy). The data acquisition and elaboration were conducted using Dropview 8400, version 3.78 software. Thermogravimetric measurements were performed on a TGA 8000 (Perkin Elmer Scientifica Italia, Milan, Italy), using a platinum crucible and setting a 10 °C/min rate from 30 to 300 °C for both complexes. *Galleria mellonella* larvae were purchased at Fishing & Adventure S.r.l. (Parma, Italy).

### 3.2. Preparation of the Chelators and the Complexes

2,2′-(((*R*, *S*)-propane-1,2-diylbis(azanediyl))bis(methylene))diphenol (**L1**). 2-hydroxybenzaldehyde (384 μL, 3.6 mmol) and (*R*, *S*)-1,2-diaminopropane (153 μL, 1.8 mmol) were mixed with few drops of acetic acid in MeOH (15 mL) before the system was put to reflux. Initially, the reaction mixture was clear and yellow, while after 3 h it visibly turned to orange with no precipitate. At that point, the system was cooled to Rt and sodium borohydride (777 mg, 20.5 mmol) was gradually and carefully added, causing the formation of hydrogen bubbles. Almost instantly, the solution turned light yellow and was then quenched with distilled water. MeOH was removed via rotary evaporation, and a saturated solution of ammonium chloride was added, followed by extraction repeated three times with DCM. The pale-yellow organic phase was dried with sodium sulfate, and the solvent was then removed, giving a dark yellow slurry. After several passages of trituration with diethyl ether, it was put in methanol and precipitated as a yellow–white cloudy solid (49%). Anal. Calcd. for C_18_H_26_N_2_O_3_ (**L1**·CH_3_OH): C, 67.90; N, 8.80; H, 8.23. Exp.: C, 67.58; N, 8.77; H, 7.39. IR (ATR, cm^−1^): 3316, 3285, 3044, 3014, 2967, 2926, 2900, 2852, 2825, 2727, 2640, 2610, 2565 (OH, NH, CH), 1610, 1590 (CC ar.), 1455 (CH bending). ESI-MS *m*/*z* (%): 287 ([M − H]^+^, 100), 327 ([M − K]^+^, 50). ^1^H-NMR (400 MHz, DMSO-*d*_6_): [ppm] 7.12–6.93 (m, 4H, CH ar.), 6.76–6.59 (m, 4H, CH ar.), 3.88–3.74 (q, 4H, CH_2_ bz.), 2.74 (m, 1H, CH), 2.58–2.45 (m, 2H, CH_2_ al.), 1.03 (d, 3H, CH_3_). ^13^C-NMR (101 MHz, DMSO-*d*_6_): [ppm] 157.2, 128.6, 127.8, 124.6, 118.5, 115.3, 53.7, 51.3, 50.4, 47.8, 18.1. (See Figures S1–S4). T_m_: 75 °C. Emission [nm]: 298 (see Figure S5). XRD structure was determined as described in Section 3.7.
2,2′-((1,2-phenylenebis(azanediyl))bis(methylene))diphenol (**L2**). 2-hydroxybenzaldehyde (192 μL, 1.8 mmol) and o-phenylenediamine (101 mg, 0.9 mmol) were mixed with few drops of acetic acid in MeOH (10 mL) before the system was put to reflux. It instantly started to form an orange precipitate. The refluxing system was cooled to Rt and sodium borohydride was gradually and carefully added (350 mg, 9 mmol), causing the formation of hydrogen bubbles. The solution turned yellow and opaque, and the reaction was then quenched with distilled water. After having removed methanol with rotary evaporation, a saturated solution of ammonium chloride was then added. The solution was then put at 4 °C overnight, giving a yellow precipitate, which was then filtered and dried at the vacuum line (93%). Anal. Calcd. for C_20_H_26_N_2_O_5_ (**L2**·3H_2_O): C, 64.15; N, 7.48; H, 7.00. Exp.: C, 64.55; N, 7.51; H, 6.02. IR (ATR, cm^−1^): 3603, 3526, 3367, 3323, 3291, 3058, 2990, 2893, 2717 (NH, CH, OH), 1595, 1566 (CC ar.), 1457 (CH bending). ESI-MS *m*/*z* (%): 321 ([M − H]^+^, 100), 343 ([M − Na]^+^, 12). ^1^H-NMR (400 MHz, DMSO-*d*_6_): [ppm] 7.16 (d, 2H, CH ar.), 7.00 (t, 2H, CH ar.), 6.77 (t, 2H, CH ar.), 6.65 (m, 2H, CH ar.), 6.46 (s, 4H, CH ar.), 4.18 (s, 4H, CH_2_). ^13^C-NMR (101 MHz, DMSO-*d*_6_): [ppm] 156.7, 136.5, 128.3, 127.5, 125.9, 120.4, 117.3, 115.5, 114.1, 110.3, 42.7 (see Figures S6–S9). T_m_: 140 °C. Emission [nm]: 429 (see Figure S10).
**C1**. 2,2′-((propane-1,2-diylbis(azanediyl))bis(methylene))diphenol (**L1**; 117 mg, 0.37 mmol) was dissolved in methanol (5 mL) at Rt. MnCl_2_·4H_2_O (99 mg, 0.50 mmol) was then added to the solution, and the mixture instantly turned black. The system is left stirring at Rt covered but not sealed for 4 days. Afterwards, it was put at 4 °C, then concentrated at the rotary evaporator, and finally, some diethyl ether was added, giving brown/black precipitate, which was then centrifuged and triturated with diethyl ether. Everything was then redissolved in dichloromethane to remove metal salt in excess, which remained as precipitate and was removed via filtration. The liquid was collected and triturated with diethyl ether, yielding a dark brown solid. (45%). Anal. Calcd. for C_17_H_29_Cl_2_MnN_2_O_6_ (**C1**·4H_2_O): C, 42.25; N, 5.80; H, 6.05. Exp.: C, 40.84; N, 5.42; H, 5.04. IR (ATR, cm^−1^): 3500, 3444, 3311, 3191, 3058, 2964, 2923, 2852, 2725 (OH, NH, CH), 1601, 1545 (CC ar.). ESI-MS *m*/*z* (%): 337 ([**L1** − Mn]^+^, 78). UV–vis *, λ_max_ [nm] (ε [cm^−1^mol^−1^L]): 276 (3000), 309 (2400), 390 (950), 449 (313), 493 (150), 566 (61). (see Figures S11–S13). T_m_: 100–110 °C. Emission [nm]: 410 (see Figure S14).

* Values obtained via deconvolution with Fityk 1.3.1^®^ program (see details in Supporting Information).

**C2**. 2,2′-((1,2-phenylenebis(azanediyl))bis(methylene))diphenol (**L2**; 140 mg, 0.41 mmol) was dissolved in methanol (6 mL) at RT. MnCl_2_·4H_2_O (87 mg, 0.44 mmol) was then added to the solution, and the mixture instantly turned black. The system was left stirring at Rt covered but not sealed for 5 days. Afterward, the solvent was removed, and the solid deposit was redissolved with dichloromethane. The dark particulate was removed via filtration, and the solvent was removed from the collected liquid phase and triturated with diethyl ether, yielding a dark brown solid. (66%). Anal. Calcd. for C_20_H_23_Cl_2_MnN_2_O_4_ (**C2**·2H_2_O): C, 49.92; N, 5.82; H, 4.82. Exp.: C, 49.80; N, 5.83; H, 3.70. IR (ATR, cm^−1^): 3670, 3508, 3450, 3191, 3064, 2982, 2902, 2717 (NH, CH, OH), 1601, 1577, 1533 (CC ar.), 1460 (CH bending). ESI-MS *m*/*z* (%): 369 ([**L2** − Mn]^+^, 90), 444 ([**L2** − Mn − Cl_2_]^+^, 5). UV–vis *, λ_max_ [nm] (ε [cm^−1^mol^−1^L]): 278 (13730), 335 (10260), 436 (4085), 522 (577), 566 (247), 616 (153) (see Figures S15–S17). T_m_: 80–100 °C. Emission [nm]: 437 (see Figure S18).

* Values obtained via deconvolution with Fityk 1.3.1^®^ program (see details in Supporting Information).

### 3.3. Stability Determination

The compounds were dissolved in PBS buffer with 1% DMSO, and their UV–visible spectra were taken with a Thermo Fisher Scientific Evolution 260 Bio Spectrophotometer (Waltham, MA, USA) in a 700 µL quartz cuvette with a path length of 1 cm. The compounds were incubated in a standardized chamber with a temperature fixed at 37 °C at atmospheric pressure.

### 3.4. UV–Visible Characterization

The compounds were dissolved at known concentrations in DMSO, and their UV–visible were taken with a Thermo fisher Scientific Evolution 260 Bio Spectrophotometer in a 3 mL quartz cuvette with a path length of 1 cm. Deconvolution of the spectra into the single absorption bands was performed via Fityk 1.3.1 software. For further details, check Supporting Information.

### 3.5. NMR Characterization

Paramagnetic NMR was taken with a Bruker Anova spectrometer at 400 MHz (Billerica, MA, USA), with chemical shift reported in δ units (ppm). NMR spectra were referenced relative to residual NMR solvent peaks. The solvent used in the spectra acquisitions is DMSO-*d*_6_.

The sequence used for the spectra was a common use ^1^H-NMR (zg30 by Bruker, Billerica, MA, USA) on which few changes were operated: **C1** (negative): SW 100 ppm, O1P −50 ppm, NS 150; **C1** (positive): SW 200 ppm, O1P 6.35 ppm, NS 100 (zoom on the downfield region); **C2** (positive): SW 600 ppm, O1P −31 ppm, NS 150; **C2** (negative): SW 60 ppm, O1P 41 ppm, NS 100.

### 3.6. Potentiometric Studies

The potentiometric titrations of **L1** and **L2** were carried out in aqueous solution at T = 25.0 ± 0.1 °C and I = 0.1 mol/L (KCl) under N_2_ stream, using 1.5 mL sample volumes. Titrations were carried out using a Metrohm OMINS automatic titrator. A Metrohm semi-micro electrode was used, which was calibrated in terms of [H^+^] by titrating HCl solutions with a KOH standardized solution. The electrode parameters were determined using the Gran’s method [37], and the pK_w_ value resulted to be 13.76 (1). Protonation studies of **L1** and **L2** were carried out by alkalimetric titrations in the pH range of 3.0–11.5 (3 samples; C_ligand_ = 0.3 mmol/L). The protonation constants of **L1** were calculated with HyperQuad 2013 [38], and the results were used to draw the species distribution curves (check Figure S19 of the Supporting Information) with the software Hyss2009 [39].

### 3.7. Single Crystal XRD

#### **Compound L1** 

Ligand **L1** crystallized form methanol and crystals suitable for single-crystal X-ray diffraction experiments were obtained. A specimen of size 0.02 × 0.02 × 0.08 mm was used for the data collection. The molecule crystallizes in space group P2_1_/c, and the asymmetric unit is formed by ligand L1 and a methanol crystallization molecule. A summary of data collection and structure refinement for the reported structures is reported in Table S2. Single crystal data were collected with Bruker D8 equipped with a PhotonII area detector (Cu Kα: λ = 1.54184 Å). The intensity data were integrated from several series of exposure frames, collected at 200 K, covering the sphere of reciprocal lattice (Bruker, (2012). APEXIV. Bruker AXS Inc., Madison, WI, USA). An absorption correction was applied using the program SADABS [40]. The structures were solved with the ShelXT [41] structure solution program using Intrinsic Phasing and refined with ShelXL [42] on F2 with full-matrix least squares using the Olex2-1.5 software [43]. Non-hydrogen atoms were refined with anisotropic thermal parameters. Hydrogen atoms were placed in their calculated positions.

CCDC deposition number: 2367089

For further details, check Supporting Information: Table S3 and Figure S20.

### 3.8. Antioxidant Activity Towards DPPH, H_2_O_2_, O_2_^·−^, and OH^·^ Monitored by Electronic Spectroscopy [21,25,33]

#### 3.8.1. DPPH

The DPPH scavenging activity was studied using a colorimetric method. Solutions of 200 μmol/L of the complexes (**C1**, **C2**) and ligands (**L1**, **L2**) were prepared in methanol. A 100 μmol/L solution of DPPH was also prepared in the same solvent. Different ratios (1 to 2.5, 1.5, 1, 0.5, 0.1) of DPPH and each complex were obtained by mixing known quantities of stock solutions to a total 1 mL volume and were incubated for 80 min at room temperature (Rt), sheltered from light. UV–visible readings were carried out at 517 nm. Maximum complex: DPPH ratio of 2:1. The percentage of radical scavenging activity (RSA %) was calculated according to Equation (1):

(1) $$RSA\%=\frac{\left(A_{sample}-A_{DPPH\cdot }\right)}{\left(A_{DPPH}-A_{DPPH\cdot }\right)}\cdot 100$$

where *A_DPPH_·* is DPPH radical absorbance, *A_DPPH_* is DPPH molecular absorbance, and *A_sample_* is the mixture absorbance. *A_DPPH_* is obtained from a DPPH solution at the same concentration in MeOH quenched with an excess of sodium ascorbate; this correction allows for a more precise value that does not underestimate the absorbance of the quenched DPPH [44].

#### 3.8.2. O_2_^·−^

Stock solutions (330 μmol/L) of the complexes (**C1**, **C2**) and KO_2_ (660 μmol/L) were prepared in dry DMSO. Superoxide concentration was evaluated using Lambert–Beer Law (ε = 2686 cm^−1^ mol^−1^ L at λ = 280 nm). UV–visible spectra of the solutions (600 μL), containing different complex-to-superoxide anion ratios: 1:2, 1:1.6, 1:1.2, 1:0.8, 1:0.4, 1:0.2.

#### 3.8.3. OH^·^

A stock solution was prepared for each complex (**C1**, **C2**) in PBS buffer (150 μmol/L, 1% DMSO). 150 μL of a 1 mmol/L solution of FeSO_4_ and 50 μL of a 10 mmol/L solution of H_2_O_2_ were diluted with 500 μL of distilled water into two distinct solutions. 100 μL of such solutions were added to 500 μL of complex stock solutions. The UV–visible spectra were recorded every 12 s for 5 min.

#### 3.8.4. H_2_O_2_

Stock solutions at 150 μmol/L of complexes (**C1**, **C2**) were prepared in PBS (with 1% DMSO), while H_2_O_2_ was diluted in distilled water to obtain a 150 mmol/L stock solution. 500 μL of complex solution was registered after the addition of 100 μL of solvent or H_2_O_2_ stock solution. UV–visible spectra were registered in the 250–750 nm window every 12 s for 5 min, plus a final spectrum at 30 min to check possible modifications.

### 3.9. Kinetic Assay of H_2_O_2_ Consumption Monitored by Clark-Type Electrode [16]

The time course of oxygen concentration was determined polarographically using a Clark-type oxygen electrode (Knick SE715 Memosens, Knick Elektronische Messgeräte GmbH & Co. KG, Berlin, Germany), plugged into a multimeter (Knick Portavo 907 Multi, Knick Elektronische Messgeräte GmbH & Co. KG, Berlin, Germany). The electrode was calibrated in open air. The reactions were carried out in DMSO/PBS 1% at standard conditions. The electrode was firstly submerged in the compound solution at known concentrations (300, 225, 150, 75, 30, 15 μmol/L; total volume: 2 mL), and afterward the 110 μL aliquot of H_2_O_2_ (stock: 9.72 mol/L; final concentration: 0.107 mol/L) was added. The oxygen concentration vs. time data were collected thanks to Paraly SW112 software version 02.02.01 package and then converted to O_2_ %. Check Table S2 for further details.

### 3.10. Kinetic Assay of H_2_O_2_ Consumption Monitored by ESI-MS [19]

ESI-MS was recorded on a Waters Acquity Ultraperformance ESI-MS spectrometer with a Single Quadrupole Detector (Waters Corporation, Milford, MA, USA). **C1** and **C2** were dissolved in CHCl_3_/MeOH 1:1 at 300 μmol/L, H_2_O_2_ was added to the vial (0.107 mol/L), and three spectra were taken at 0, 3, and 6 min.

### 3.11. CV Characterization

Cyclic voltammetry (CV) experiments were conducted using a μStat 8000 Multi Potentiostat/Galvanostat (Metrohm Italiana Srl, 21040 Origgio (VA), Italy) on screen-printed electrodes with carbon working and counter electrodes and silver pseudoreference (SPCE DRP-C110). The data analysis was performed using Dropview 8400, version 3.78 software. A 1 mmol/L ferrocene solution, prepared in a 9:1 dichloromethane/dimethylformamide solvent mixture containing 0.1 mol/L tetrabutylammonium hexafluorophosphate, exhibited an E_1/2_ potential of +0.5 V with respect to the silver pseudoreference of SPCEs.

The CV characterization of the complexes was carried out in 1 mmol/L 9:1 dichloromethane/dimethylformamide solutions containing 0.1 mol/L tetrabutylammonium hexafluorophosphate as the supporting electrolyte. To achieve this, SPCEs were immersed in the solution of the compound under investigation, and the resulting cyclic voltammograms were acquired within a potential window spanning from −1.5 to +1.25 V at a temperature of 20 °C and a scan rate of 20 mV/s.

### 3.12. Biological Assays

The A549 cells were purchased from the American Type Culture Collection (ATCC, Manassas, VA, USA) and were grown in RPMI 1640 medium containing 10% heat-inactivated fetal bovine serum, 100 U/mL penicillin, 100 μg/mL streptomycin, and 2 μmol/L l-glutamine at 37 °C with 5% CO_2_. The experiments were conducted with cells grown in a flask and in log phase for a period of no less than 24 h.

The cells were seeded into 96-well and the day after exposed to drugs (ligands and their relative complexes) at different concentrations (0.1–100 µmol/L) for 24 and 48 h. An untreated sample was used as a control. All treatments were performed in triplicate. At 24 and 48 h of compound exposure, a 3-(4,5-dimethylthiazol-2-yl)-2,5-diphenyltetrazolium bromide (MTT) colorimetric assay was added at a concentration of 0.5 mg/mL into each well and incubated at 37 °C for 3 h. MTT assay is based upon the ability of metabolically active cells to reduce MTT into formazan by the action of mitochondrial dehydrogenases. After, formazan crystals were dissolved, and the absorbance was assessed using a microwell plate reader at a wavelength of 550 nm. To confirm viability, a cell count analysis was carried out using a hemocytometer by the Trypan blue exclusion method. Half of the maximum inhibitory concentration (IC_50_) was then calculated as the concentration resulting from a 50% reduction in cell growth compared to the untreated control.

A549 cells were seeded and after 24 h treated with the drugs at the concentration of IC_50_ for 12 h and 48 h. Two untreated flasks were used as controls. At the end of the treatment, the cells, after being detached and counted, were centrifuged at 1500 rpm for 7 min.

The cells were then placed on ice and washed in PBS, resuspended with 1 mL of PBS + EDTA (0.5 mmol/L), and finally fixed with 3 mL of cold 96% ethyl alcohol.

Subsequently, after washing twice in PBS, the cells were labeled with propidium iodide (20 µg/mL in PBS) and 12.5 µg/mL of RNase-A and incubated overnight at 4 °C.

The samples thus obtained were analyzed by flow cytometry (CytoFLEX Beckman Coulter, Brea, CA, USA) and analyzed with the FlowJo software v10.10 (Ashland, OR, USA) (see Figure S21).

Caspase-Glo 3/7 assay was used to evaluate pro-apoptotic effects in accordance with manufacturer’s instructions (Promega Corporation, Madison, WI, USA). A549 cells in exponential phase of growth were cultured and seeded (1 × 10^5^ cells/mL) in 96-well culture plate and exposed to drugs for 24 h; untreated samples were used as a control. The day after A549 cells were incubated with 100 μL of Caspase-Glo 3/7 reagent at 37 °C for 30 min and then luminescence was measured by Varioskan™ LUX multimode microplate reader (Thermofisher Scientific, Waltham, MA, USA).

All treatments were performed in triplicate. Changes in caspase activity were assessed by normalizing treated to untreated cells.

The thiobarbituric acid reactive substances (TBARS) method was used to identify cellular lipid peroxidation, an important indicator of oxidative stress. This assay is based on the formation of malondialdehyde (MDA), which reacts with two equivalents of thiobarbituric acid to form a red fluorescent chromogen detectable by spectrophotometry. Briefly, 10^6^ cells were collected after 24 h from treatment with the complexes, freeze/thawed three times, and centrifuged at 3000× *g* for 10 min. The supernatant was mixed with an equal volume of 0.2 mol/L orthophosphoric acid and a 1/16 volume of thiobarbituric acid in 0.1 mol/L NaOH, then incubated for 45 min at 95 °C. N-butanol/NaCl was added for TBARS extraction. The samples were centrifuged at 3000× *g* for 2 min, and the supernatant was collected. TBARS concentration was measured using a Varioskan Lux (excitation 515 nm, emission 545 nm). Malondialdehyde was used as a standard for the calibration curve with a range of 0–10 μmol/L. TBARS concentrations were normalized for the total protein concentrations.

Protein concentrations were quantified by the bicinchoninic acid (BCA) protein assay according to the manufacturer’s microwell plate protocol, and bovine serum albumin dilutions were included as standard curves. Absorbances were read at 550 nm by Varioskan™ LUX microwell plate reader.

RNA from treated and untreated cultured cells was extracted using TRIzol reagent (Thermo Fisher Scientific), following the manufacturer’s instructions. Subsequently, to eliminate genomic DNA contamination a DNase I (DNA-free kit; Thermo Fisher Scientific, Waltham, MA, USA) was used, and the RNA concentration was determined using a Varioskan Lux.

cDNA was then synthesized with a commercial kit and analyzed by real-time PCR using specific primers, including exon–exon junctions specifically designed for heme-oxygenase 1 (HO-1), superoxide dismutase 1 (SOD-1), and superoxide dismutase 2 (SOD-2). HMGB1 and TP53 gene expression were quantified using Taqman gene expression assays (Assay ID: Hs01923466_g1 and Hs01034249_m1, respectively; Thermo fisher Scientific, Waltham, MA, USA). Data were normalized over the phosphoglycerate kinase 1 (PGK1) housekeeping gene, and the expressions were calculated as 2^−ΔΔCt^.

All data obtained from experiments performed in triplicate were expressed as the mean ± standard deviation (SD). One-way analysis of variance (ANOVA) with Dunnett’s or Tukey’s post hoc tests was used for data comparison.

### 3.13. Galleria mellonella Toxicological Assays [34,35,36]

*Galleria mellonella* larvae in the 400–500 mg range were divided into groups of 20 each and placed in large Petri plates to perform a toxicological test of the complexes **C1** and **C2** at different concentrations. The results were compared to those from clear solvent injection and untreated larvae groups to detect mortality related only to the activity of the compounds. Solutions of **C1** and **C2** in distilled water (10% DMSO) at different concentrations (1.7 mmol/L, 0.85 mmol/L, 0.43 mmol/L) were prepared. 10 μL of these solutions were injected in *Galleria mellonella* larvae using a 100 μL Hamilton syringe with a pointed needle, holding the larvae at one of the pseudopods. Considering that larvae of this size are estimated to have a 284 μL internal volume, it was considered that the toxicological tests were performed with 60 μmol/L, 30 μmol/L, and 15 μmol/L internal concentrations of compound. After the injection, the larvae were incubated at 37.4 °C, and mortality was checked every day for a week.

### 3.14. Computational Evaluation

The ground state geometry of **C1** and **C2** was optimized in DFT (CAM-B3LYP/SDD) in Gaussian [45,46]. We considered a quintet spin state and set default spin [47].

## 4. Conclusions

We synthesized two Mn(III) salen-like complexes that differ in the molecular bridge between the two amines (diaminic bridge): **C1** has an aliphatic bridge, while **C2** has an aromatic bridge.

The characterization of these complexes involved several techniques, including IR spectroscopy, ESI-MS, elemental analysis (EA), thermogravimetric analysis (TGA), electronic spectroscopy, paramagnetic NMR spectroscopy, potentiometry, and cyclic voltammetry. These analyses, along with Gaussian theoretical calculations, provided insights into their molecular structure, overall polarity, and redox properties. Our initial findings highlighted the significance of the diaminic bridge in determining the intrinsic properties of these complexes. Specifically, we observed that **C1** is likely in a *trans* configuration, whereas **C2** is plausibly *cis-β*. Additionally, **C1** is a cation while **C2** is neutral, and they exhibit differing redox properties.

Obtaining crystals suitable for diffraction analysis was challenging, likely due to the loss of hydration solvent, in our hypothesis, which causes the crystals to deteriorate. In fact, dark crystals were isolated from the mother liqueur. However, during the data collection process, the crystal deteriorated, rendering the collection useless. Different crystallization mixtures as well as varying temperatures were attempted, but with little success. An interesting challenge could lie in exploring alternative crystallization techniques.

We performed numerous studies using electronic spectroscopy to compare the efficacy of **C1** and **C2** against various reactive species, revealing differences in their abilities as ROS scavengers. Further techniques, such as kinetic oximetry and time-resolved ESI-MS analysis, demonstrated that both complexes act as catalysts, particularly in the consumption of H_2_O_2_.

Biological studies indicated that **C1** and **C2** induce apoptosis in lung cancer cells; they are non-toxic according to the *Galleria mellonella* larval model. These studies also confirmed the influence of the diaminic bridge on the properties of **C1** and **C2**, as they produced different effects in regulating ROS-limiting enzymes, ROS consumption, and other factors.

Mn(III) synzymes show significant potential as therapeutic agents by leveraging two key mechanisms: selective induction of apoptosis and the production of reactive oxygen species (ROS). These processes enable the targeted destruction of cancerous or diseased cells, minimize damage to healthy tissue, and enhance immune system activation. Despite their promise, challenges such as balancing ROS levels, optimizing targeted delivery, and integrating with combination therapies remain critical. Future research should focus on refining selectivity, improving delivery methods, and ensuring precise therapeutic control to maximize efficacy and minimize side effects.

## Figures and Tables

**Table 1:** Radical Scavenging activity of complexes and ligands against DPPH was evaluated via colorimetric method. Solutions of 200 μmol/L of the complexes (**C1**, **C2**) and ligands (**L1**, **L2**) were prepared in methanol. A 100 μmol/L solution of DPPH was also prepared in the same solvent. Different DPPH/compounds were obtained by mixing known quantities of stock solutions in total of 1 mL volume and were incubated for 80 min at RT, sheltered from light. UV–visible readings were carried out at 517 nm. In comparison, [Mn(salen)Cl] was reported with RSA: 67.8% in EtOH by Segat et al. [25].

Compound	RSA%
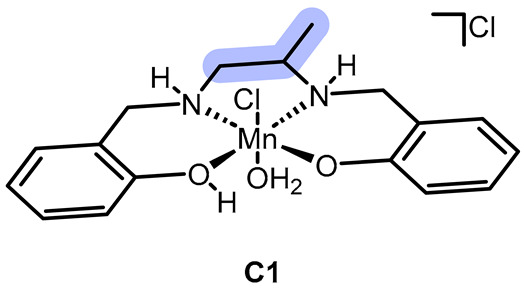	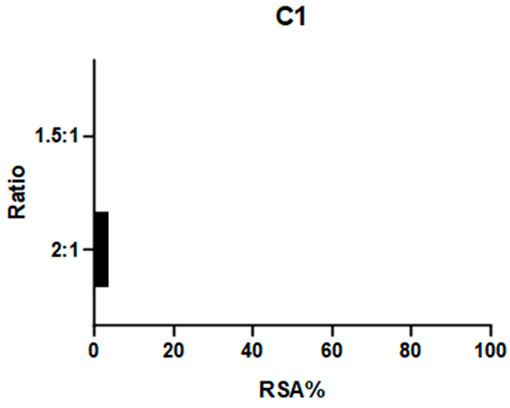
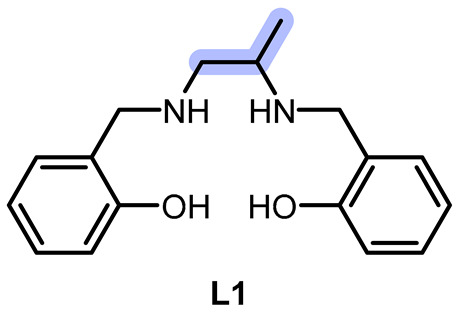	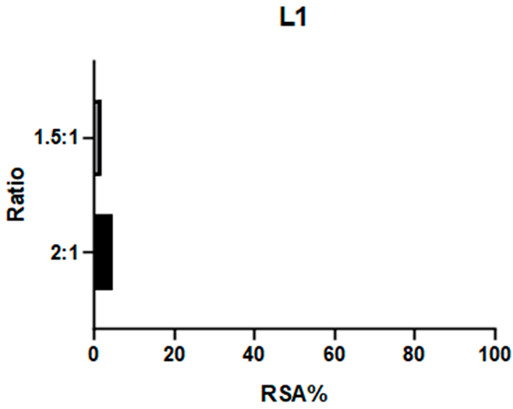
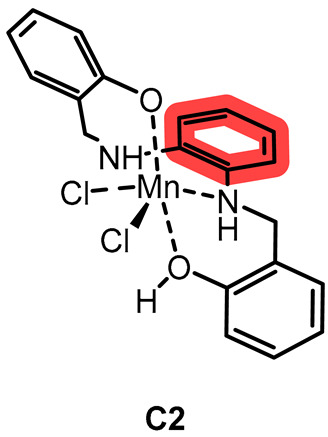	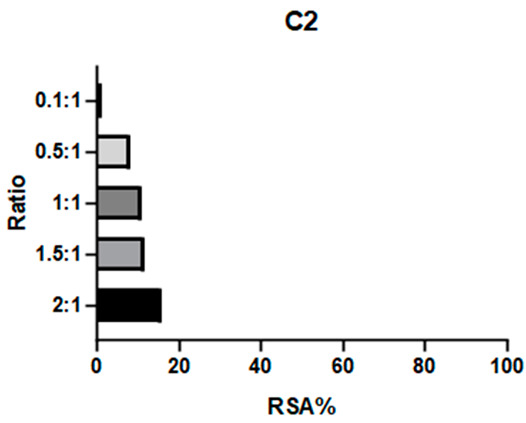
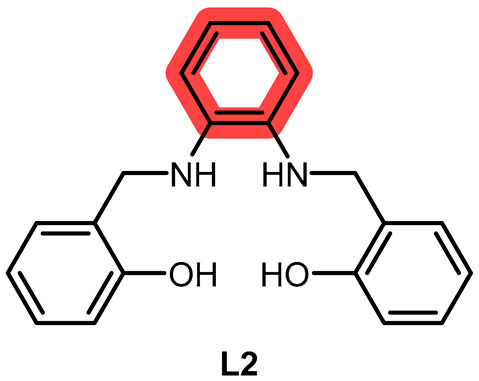	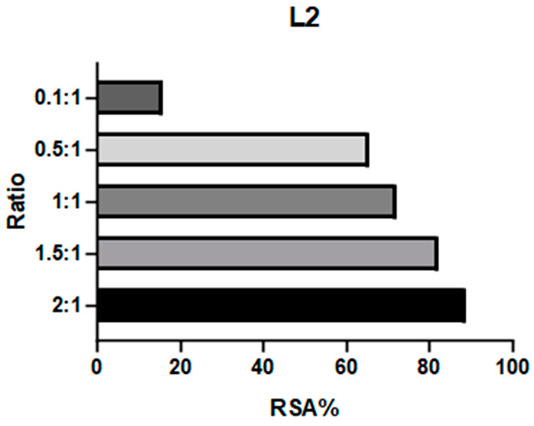

**Table 2:** Antiproliferative activity display as IC_50_ [μmol/L] value within 24 and 48 h.

	IC_50_ 24 h	IC_50_ 48 h
**L1**	>100	18 ± 2
**C1**	65 ± 3	12.3 ± 0.5
**L2**	29.2 ± 0.9	8.3 ± 0.9
**C2**	>100	24 ± 4

**Table 3:** The table shows the percentages of cells in the different phases of the cell cycle after 12–48 h of treatment with compounds. Three distinct phases could be recognized in the proliferating cell population, corresponding to different peaks: G_0_/G_1_, S, and G_2_/M phase. Values are mean ± SD of three separate experiments, each carried out in triplicate. Statistical significance is indicated as follows: ^+++^ *p* < 0.001, ^++^ *p* < 0.01, ^+^ *p* < 0.05 values vs. control; * *p* < 0.05, ** *p* < 0.01, *** *p* < 0.001 **C** vs. **L**.

	subG_0_G_1_	G_0_G_1_	S	G_2_M
CTR 12 h	1.8 ± 0.3	58 ± 1	21.3 ± 0.7	18.6 ± 0.1
**L1** 12 h	2.5 ± 0.6	72 ± 2 ^+++^	18 ± 2	7.4 ± 0.5 ^+++^
**C1** 12 h	2.9 ± 0.6	64.7 ± 0.7 ^+++^***	22.2 ± 0.8 *	10.2 ± 0.6 ^+++^
**L2** 12 h	2.3 ± 0.7	56.1 ± 0.5	24.1 ± 0.1	18 ± 1
**C2** 12 h	6.6 ± 0.8 ^+++^***	59.4 ± 0.8	20.4 ± 0.5	14 ± 1 ^+++^
CTR 24 h	2.2 ± 0.5	56.7 ± 0.6	21.5 ± 0.9	19.6 ± 0.3
**L1** 24 h	5.4 ± 0.4	69.2 ± 0.2 ^+++^	17.8 ± 0.2 ^+++^	7.6 ± 0.5 ^+++^
**C1** 24 h	6.9 ± 0.7 ^+^	63 ± 1 ^++^**	21.1 ± 0.8 ***	8.7 ± 0.8 ^+++^
**L2** 24 h	5.6 ± 0.6	57 ± 1	21.6 ± 0.5	15.9 ± 0.6 ^+++^
**C2** 24 h	26 ± 3 ^+++^***	53 ± 3	14.6 ± 0.5 ^+++^***	6.0 ± 0.4 ^+++^***
CTR 48 h	1.3 ± 0.3	67 ± 1	16.4 ± 0.9	15.2 ± 0.3
**L1** 48 h	18 ± 1 ^+++^	62 ± 2 ^+^	13.0 ± 0.8 ^+^	10 ± 10 ^+++^
**C1** 48 h	24.0 ± 0.5 ^+++^***	54.0 ± 0.3 ^+++^**	14 ± 1	7.6 ± 0.5 ^+++^
**L2** 48 h	7 ± 1 ^+++^	58 ± 1 ^+++^	18 ± 2	16.4 ± 0.5
**C2** 48 h	39 ± 1 ^+++^***	40 ± 2 ^+++^***	14.9 ± 0.3 *	7 ± 1 ^+++^***

## Data Availability

The data presented in this study are available in the article or Supplementary Material.

## Supplementary Materials

The following supporting information can be downloaded at: https://www.mdpi.com/article/10.3390/ijms26010150/s1.
