# Supplementary material for: Cytotoxic ROS-Consuming Mn(III) Synzymes: Structural Influence on Their Mechanism of Action
Source: Int J Mol Sci. 2024 Dec 27;26(1):150. doi: 10.3390/ijms26010150 (PMC11719908; doi:10.3390/ijms26010150)
Supplement: Supplementary file 1 [file ijms-26-00150-s001.zip › ijms-3393849-supplementary.pdf]

*Article*

# Cytotoxic ROS-consuming Mn(III) Synzymes: Structural Influence on their Mechanism of Action.

**Lorenzo Verderi[1], Niccolò Nova[1], Valentina Borghesani[1], Matteo Tegoni[1], Marco Giannetto[1], Simone Fortunati[1], Luca Ronda[2], Silvana Pinelli[2], Paola Mozzoni[2,3], Maria Nicastro[2], Benedetta Ghezzi[2], Giorgio Pelosi[1,3], Franco Bisceglie[1,3,*]**

[1] Department of Chemistry, Life Sciences and Environmental Sustainability, University of Parma, 43124 Parma, Italy; lorenzo.verderi@unipr.it (L.V.); niccolo.nova@studenti.unipr.it (N.N.); valentina.borghesani@unipr.it (V.B.); matteo.tegoni@unipr.it (M.T.); marco.giannetto@unipr.it (M.G.); simone.fortunati@unipr.it (S.F.); giorgio.pelosi@unipr.it (G.P.); franco.bisceglie@unipr.it (F.B.);

[2] Department of Medicine and Surgery, University of Parma, Via Volturno, 39, 43125, Parma, Italy; luca.ronda@unipr.it (L.R.); silvana.pinelli@unipr.it (S.P.); paola.mozzoni@unipr.it (P.M.); maria.nicastro@unipr.it (M.N.); benedetta.ghezzi@unipr.it (B.G.);

[3] Centre of Excellence for Toxicological Research (CERT), University of Parma, 43124 Parma, Italy; paola.mozzoni@unipr.it (P.M.); giorgio.pelosi@unipr.it (G.P.); franco.bisceglie@unipr.it (F.B.);

## Supporting Information

### Ligands Characterization Spectra

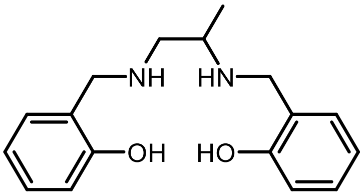


L1

IR

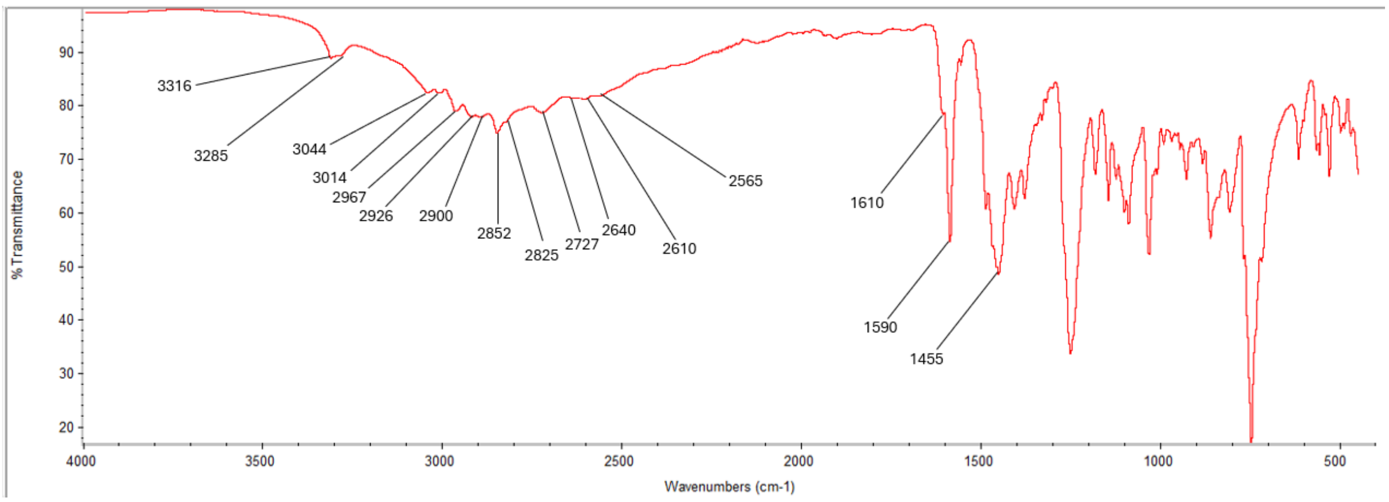


**Figure S1**

IR (ATR, $cm^{-1}$): 3316, 3285, 3044, 3014, 2967, 2926, 2900, 2852, 2825, 2727, 2640, 2610, 2565 (OH, NH, CH), 1610, 1590 (CC ar.), 1455 (CH bending).

ESI-MS

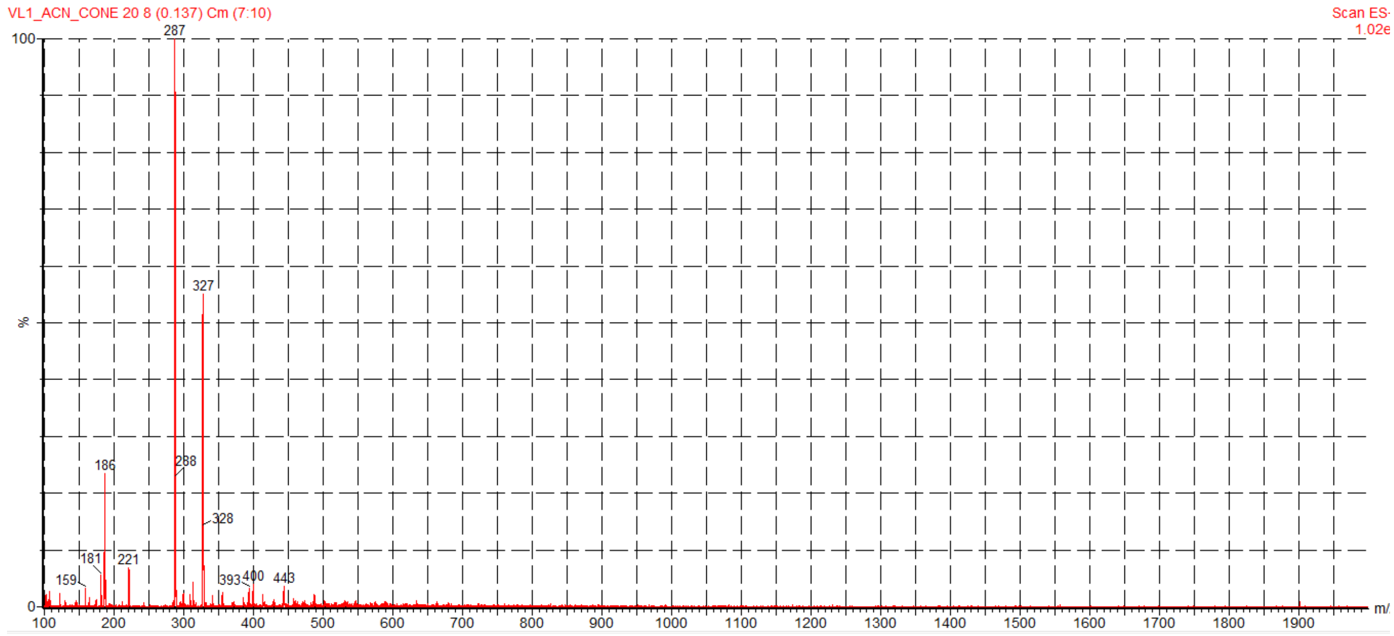


**Figure S2**

ESI-MS m/z (%): 287 ([M-H]+, 100), 327 ([M-K]+, 50).

$^{1}$H-NMR

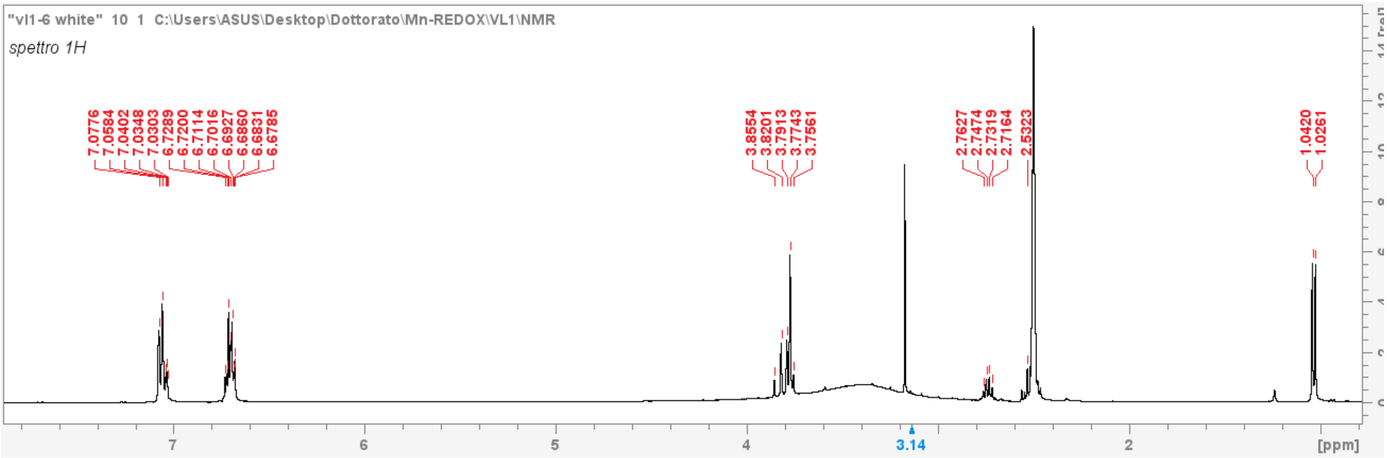


**Figure S3**

$^{1}$H-NMR (400 MHz, DMSO-d$_6$): [ppm] 7.12-6.93 (m, 4H, CH ar.), 6.76-6.59 (m, 4H, CH ar.), 3.88-3.74 (q, 4H, CH$_2$ bz.), 2.74 (m, 1H, CH), 2.58-2.45 (m, 2H, CH$_2$ al.), 1.03 (d, 3H, CH$_3$).

$^{13}$C-NMR

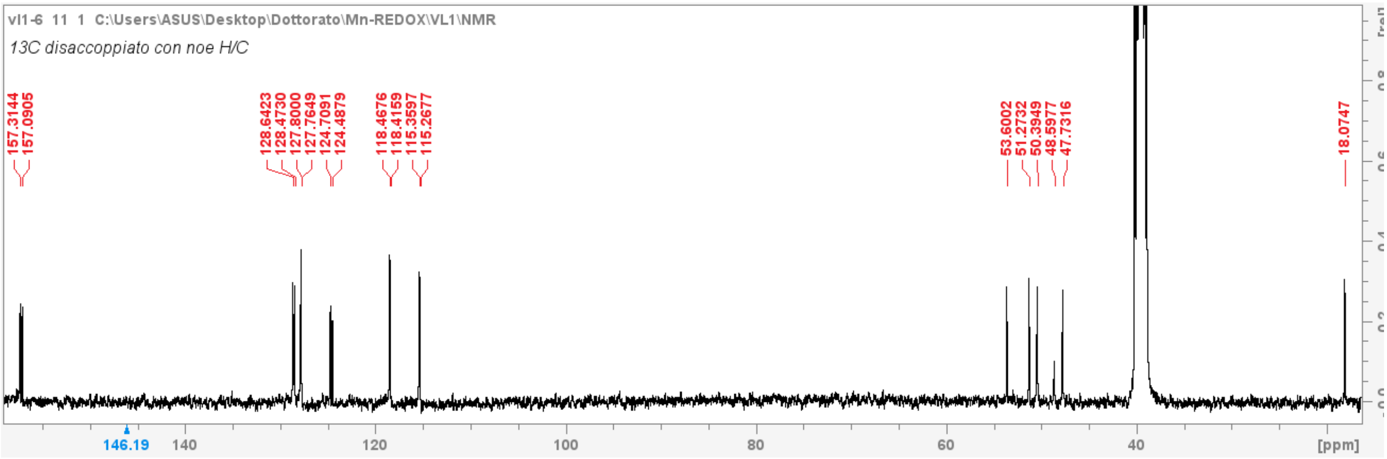


**Figure S4**

$^{13}$C-NMR (101 MHz, DMSO-d$_6$): [ppm] 157.2, 128.6, 127.8, 124.6, 118.5, 115.3, 53.7, 51.3, 50.4, 47.8, 18.1.

Fluorescence

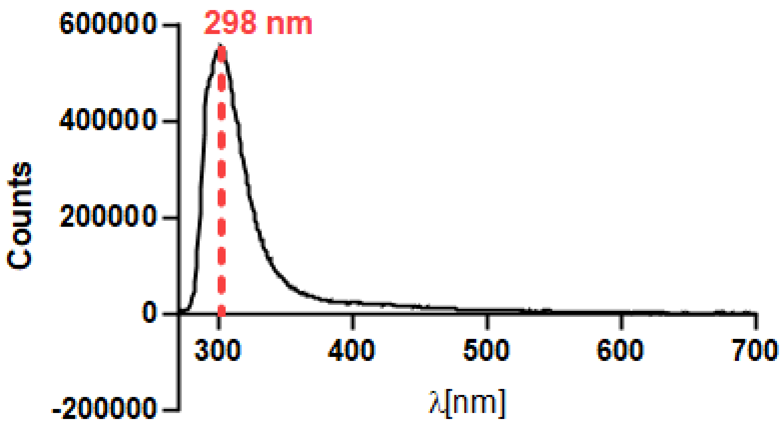


**Figure S5**

Emission [nm]: 298.

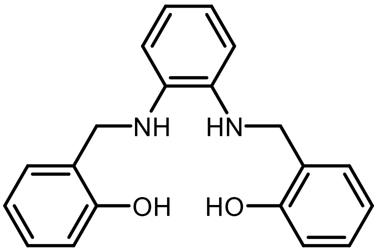


**L2**

IR

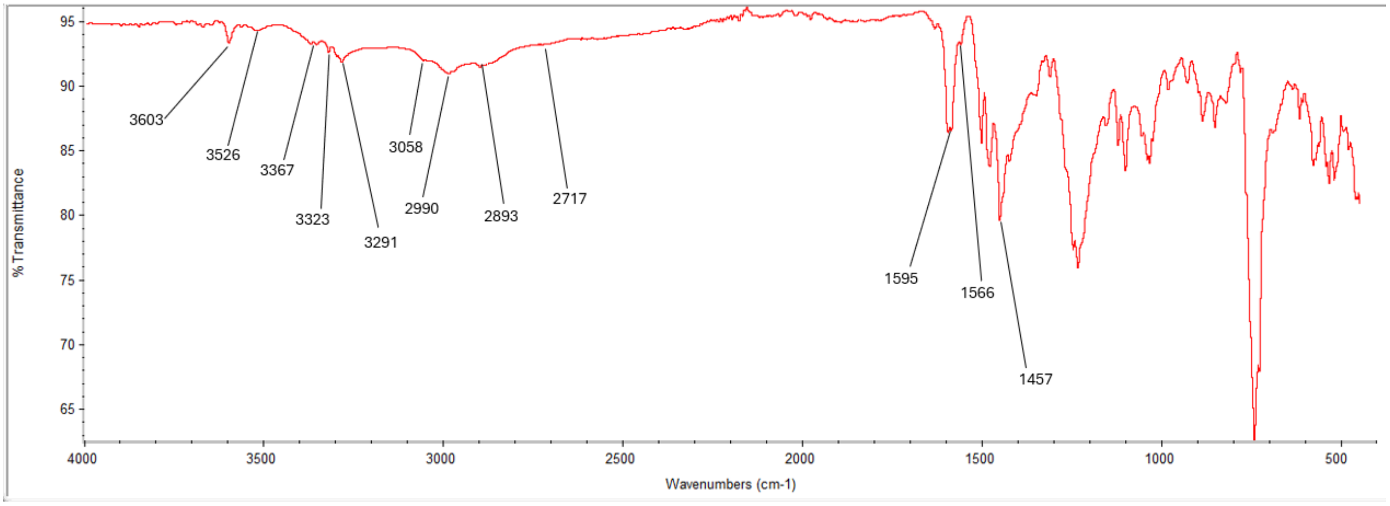


**Figure S6**

IR (ATR, $cm^{-1}$): 3603, 3526, 3367, 3323, 3291, 3058, 2990, 2893, 2717 (NH, CH, OH), 1595, 1566 (CC ar.), 1457 (CH bending).

ESI-MS

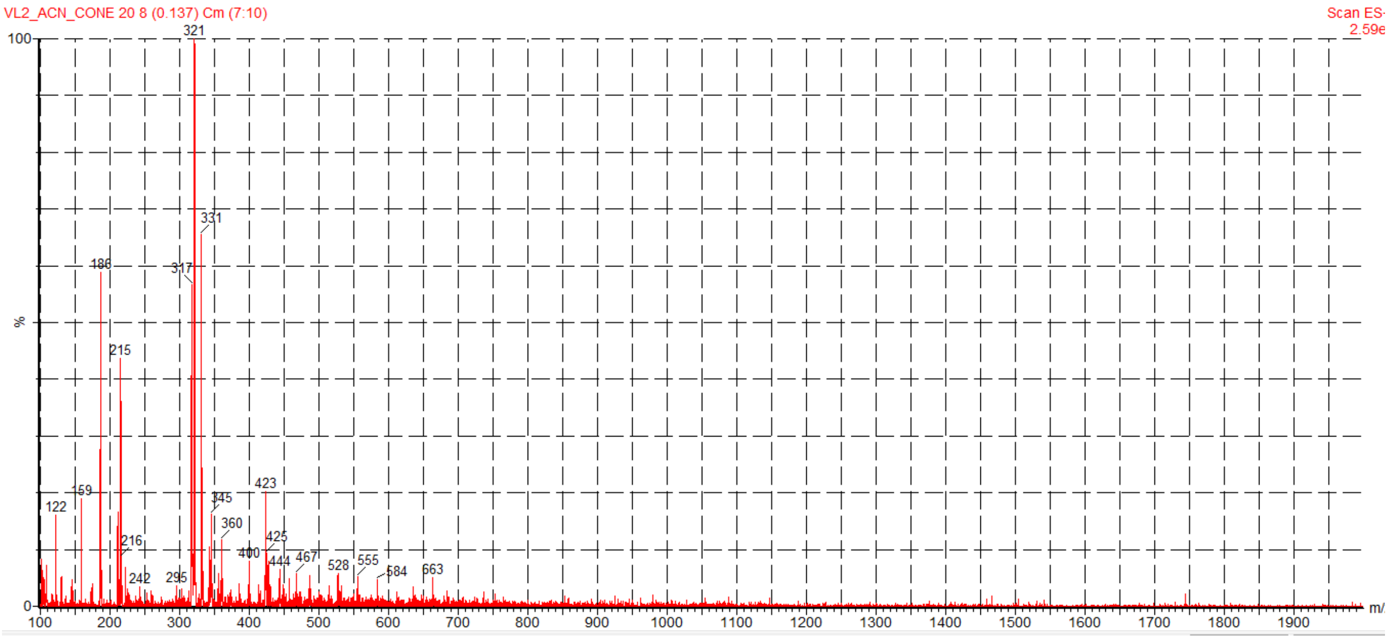


**Figure S7**

ESI-MS m/z (%): 321 ([M-H]$^+$, 100), 343 ([M-Na]$^+$, 12).

$^1$H-NMR

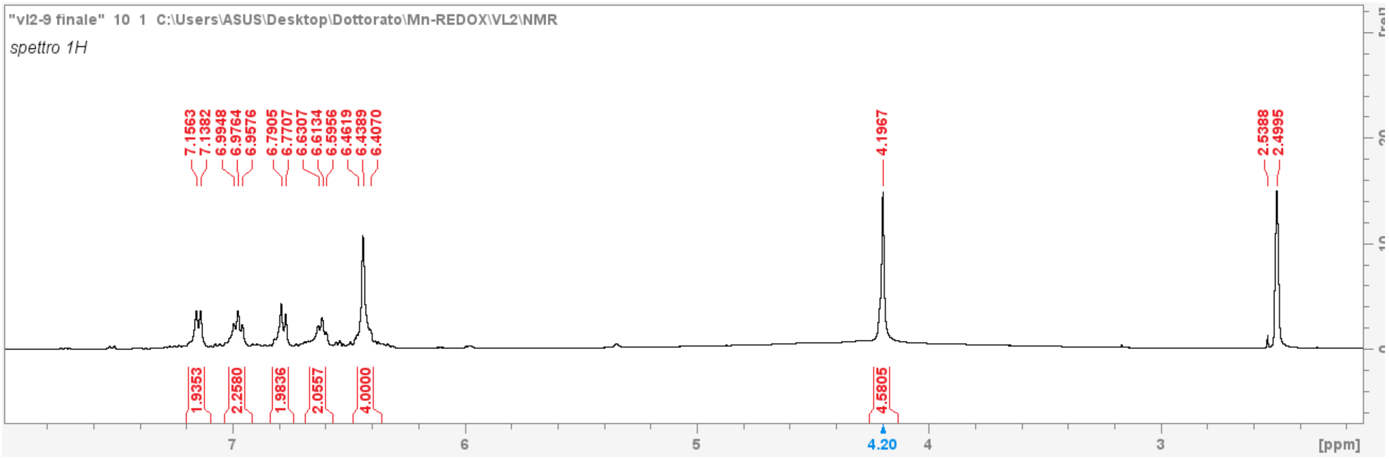


**Figure S8**

$^1$H-NMR (400 MHz, DMSO-$d_6$): [ppm] 7.16 (d, 2H, CH ar.), 7.00 (t, 2H, CH ar.), 6.77 (t, 2H, CH ar.), 6.65 (m, 2H, CH ar.), 6.46 (s, 4H, CH ar.), 4.18 (s, 4H, $CH_2$).

$^{13}$C-NMR

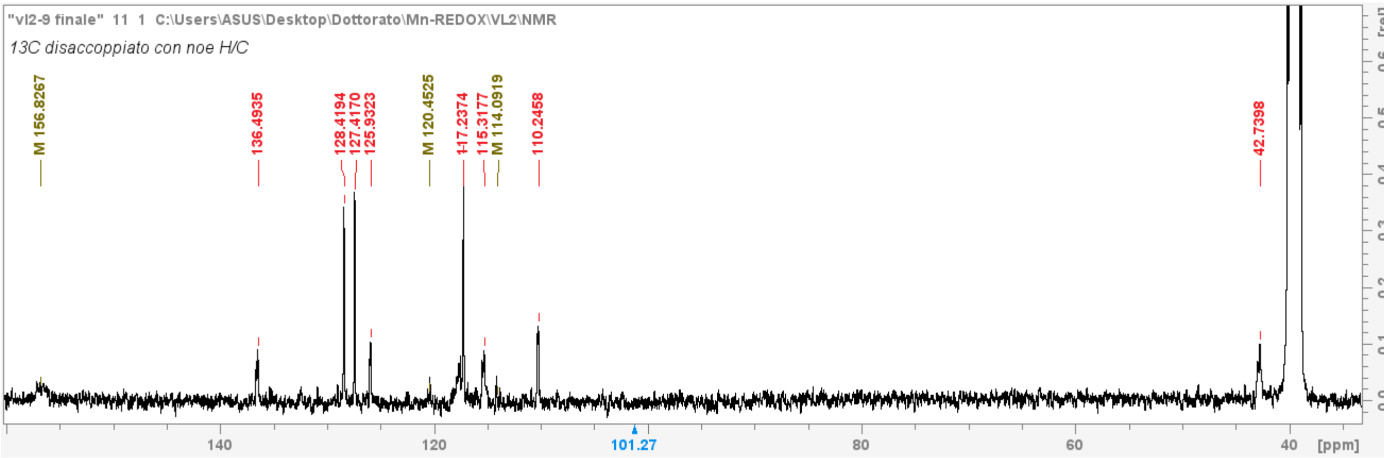


**Figure S9**

$^{13}$C-NMR (101 MHz, DMSO-$d_6$): [ppm] 156.7, 136.5, 128.3, 127.5, 125.9, 120.4, 117.3, 115.5, 114.1, 110.3, 42.7.

Fluorescence

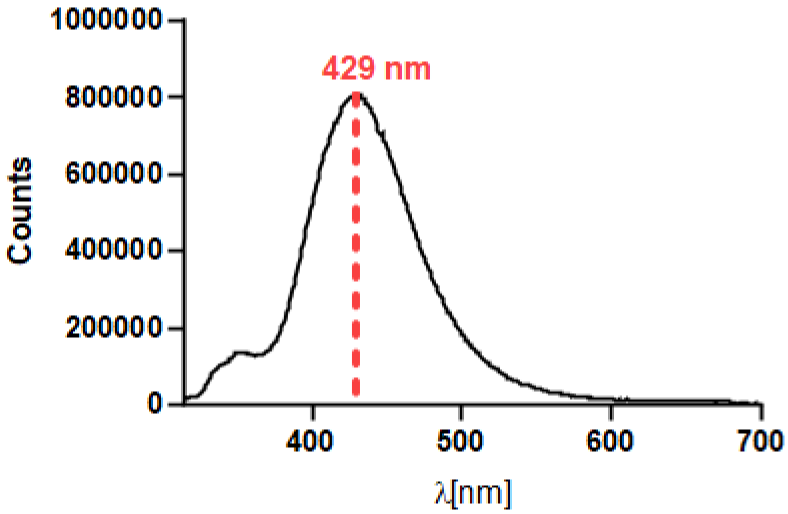


**Figure S10**

Emission [nm]: 429.

**Complexes Characterization Spectra**

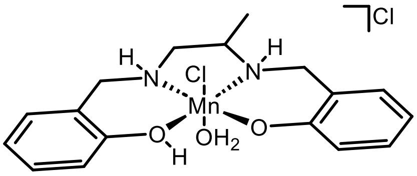


**C1**

IR

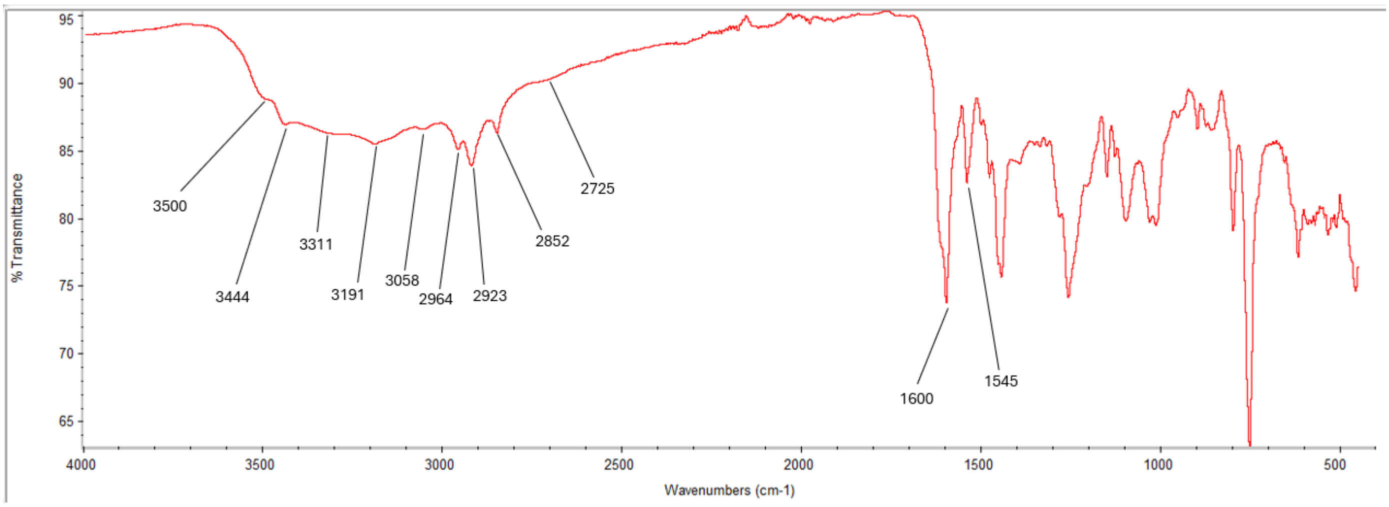


**Figure S11**

IR (ATR, $cm^{-1}$): 3500, 3444, 3311, 3191, 3058, 2964, 2923, 2852, 2725 (OH, NH, CH), 1601, 1545 (CC ar.).

ESI-MS

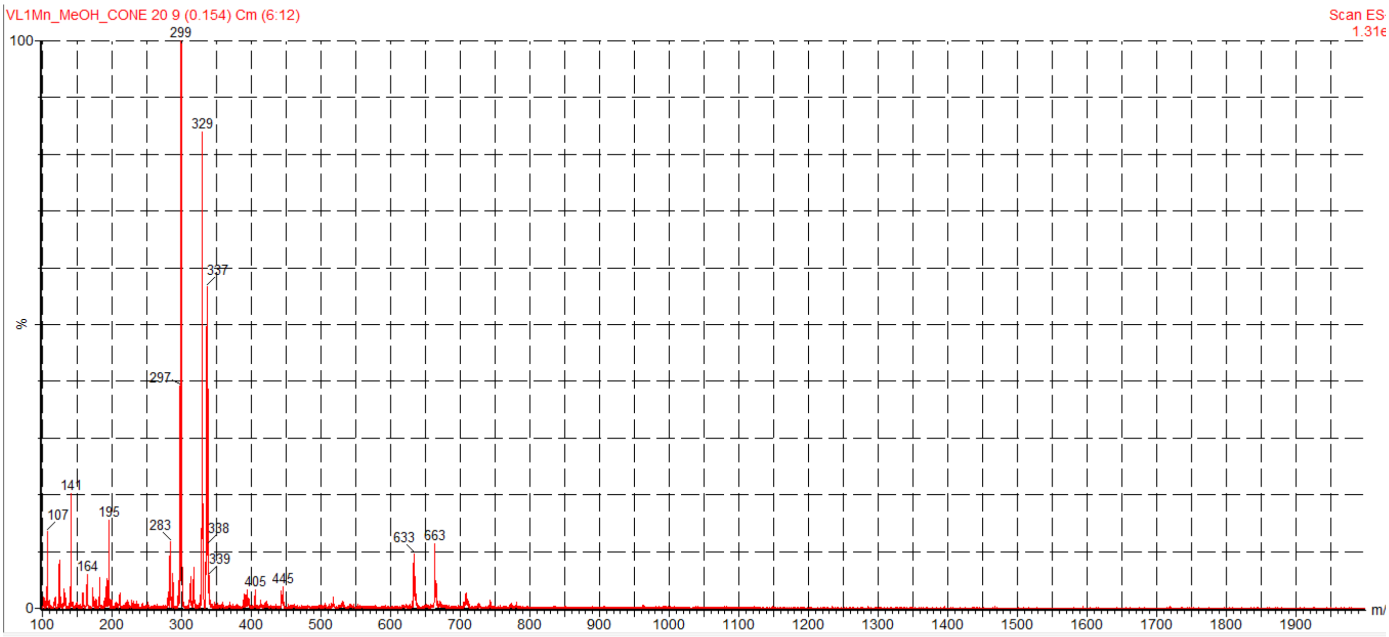


**Figure S12**

ESI-MS m/z (%): 337 ($[L1-Mn]^+$, 78).

Electronic Spectrum Deconvolution

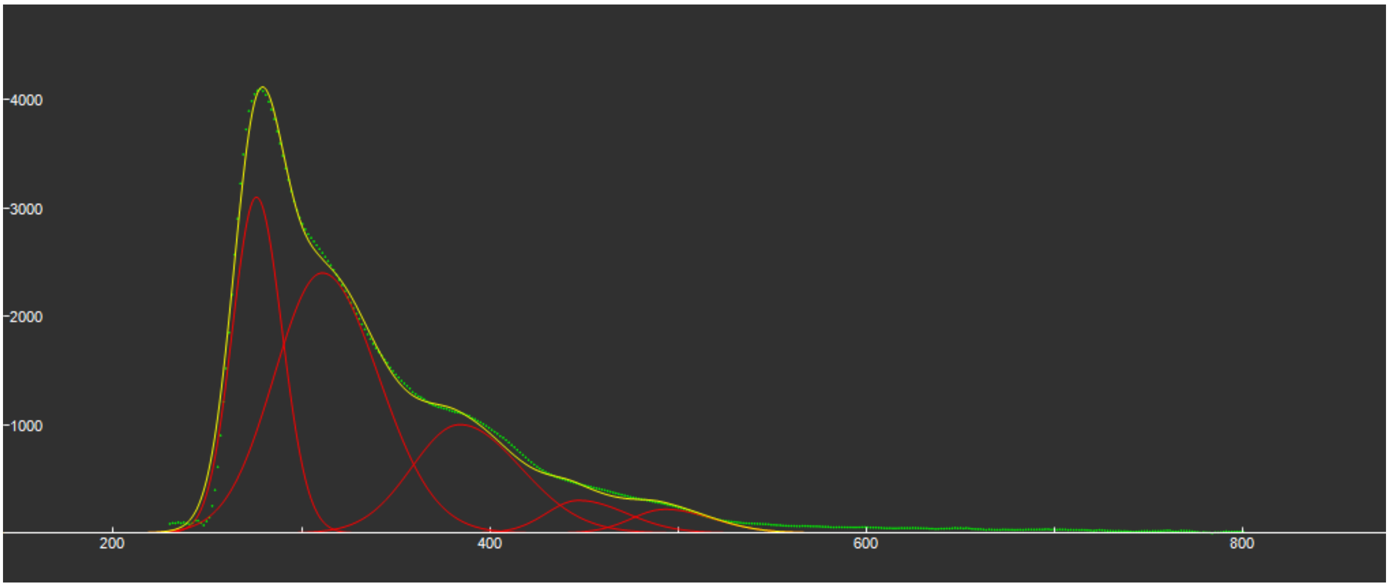


**Figure S13**

UV-vis, $\lambda_{max}$ [nm] ($\varepsilon$ [$cm^{-1}mol^{-1}L$]): 276 (3000), 309 (2400), 390 (950), 449 (313), 493 (150), 566 (61).

Fluorescence

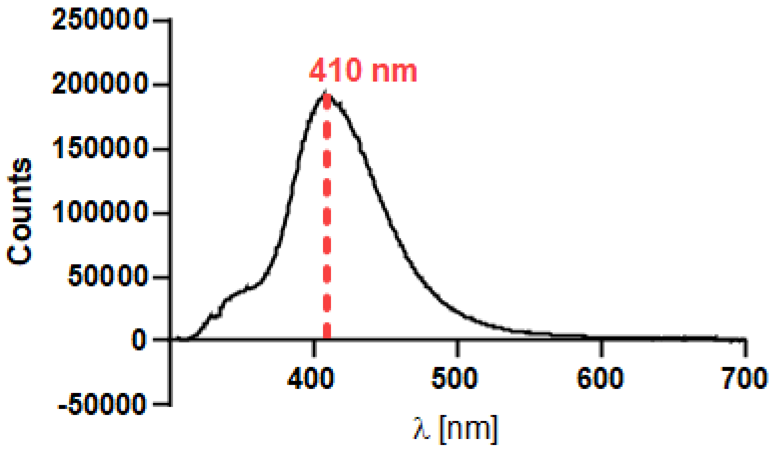


**Figure S14**

Emission [nm]: 410.

Thermogravimetric Analysis

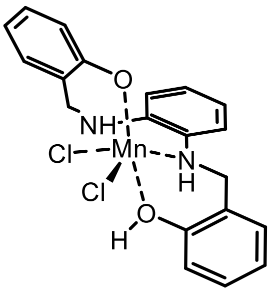


**C2**

IR

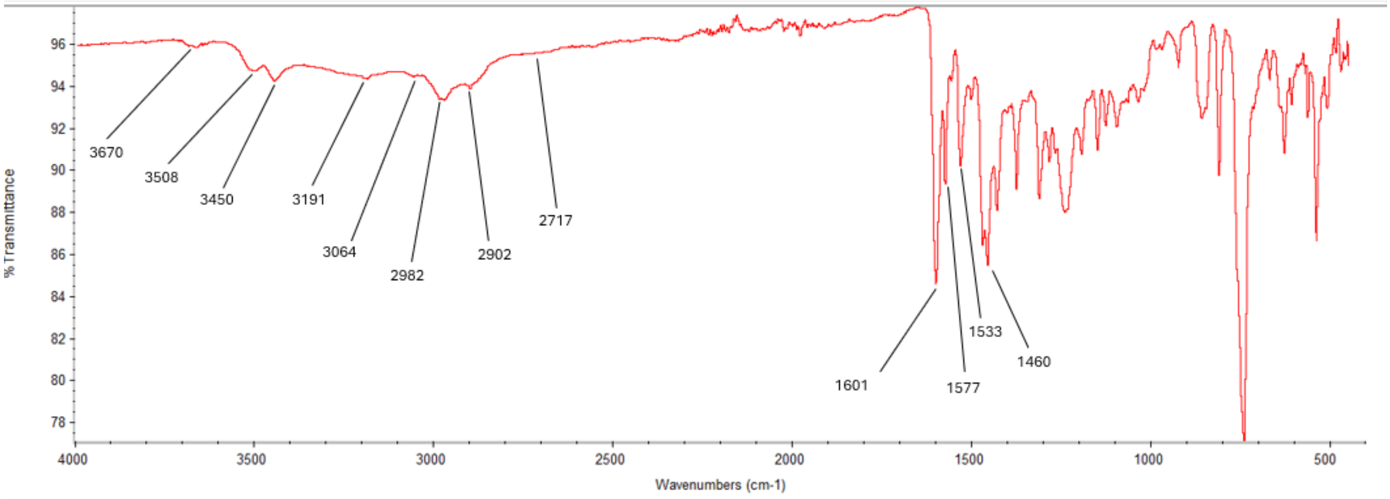


**Figure S15**

IR (ATR, $cm^{-1}$): 3670, 3508, 3450, 3191, 3064, 2982, 2902, 2717 (NH, CH, OH), 1601, 1577, 1533 (CC ar.), 1460 (CH bending).

ESI-MS

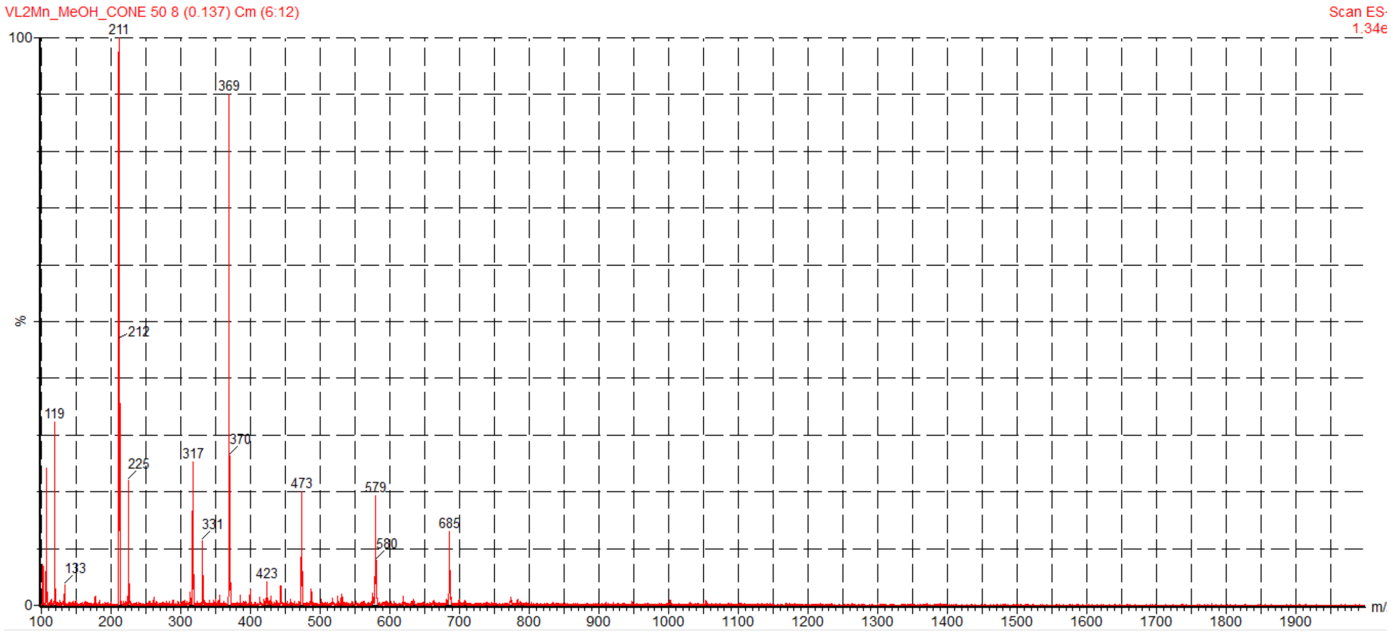


**Figure S16**

ESI-MS m/z (%): 369 ([**L2**-Mn]$^+$, 90), 444 ([**L2**-Mn-$Cl_2$]$^+$, 5).

Electronic Spectrum Deconvolution

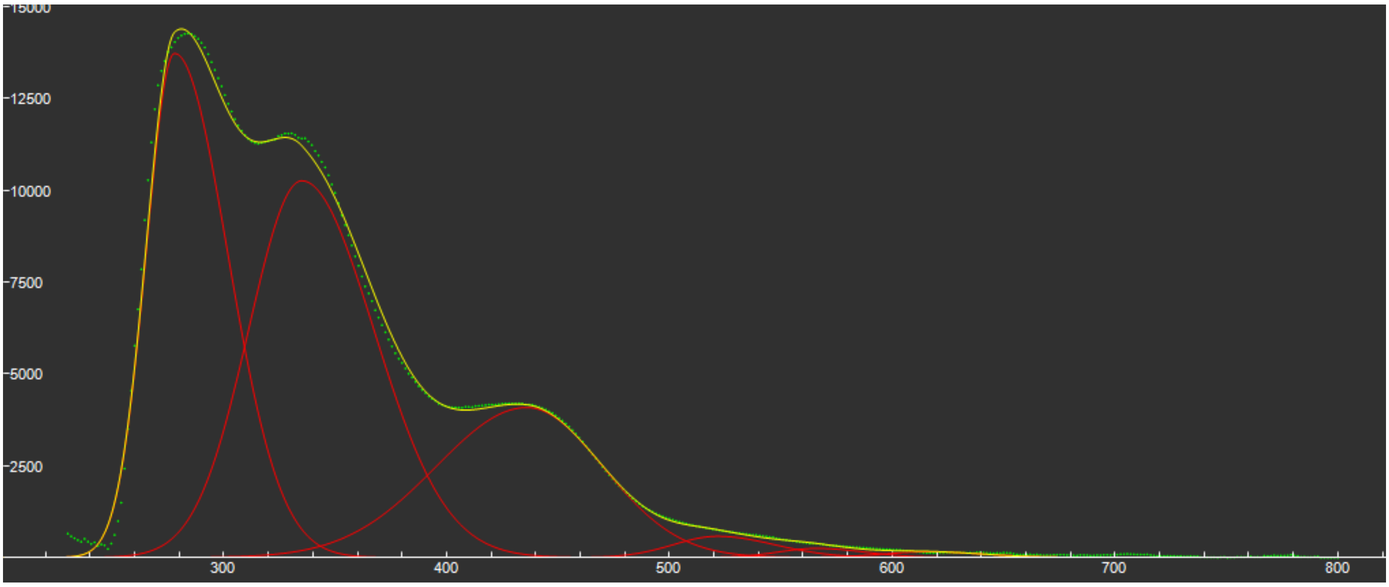


**Figure S17**

UV-vis, $\lambda_{max}$ [nm] (ε [$cm^{-1}mol^{-1}L$]): 278 (13730), 335 (10260), 436 (4085), 522 (577), 566 (247), 616 (153).

Fluorescence

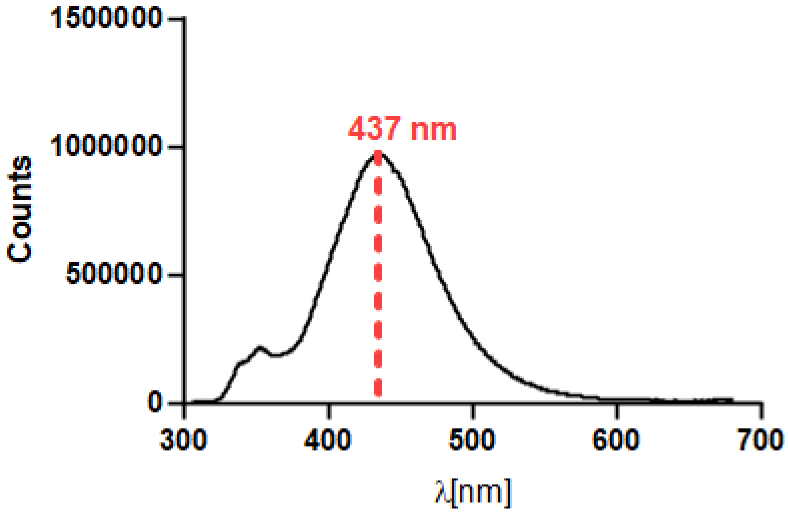


**Figure S18**

Emission [nm]: 437.

Voltammograms

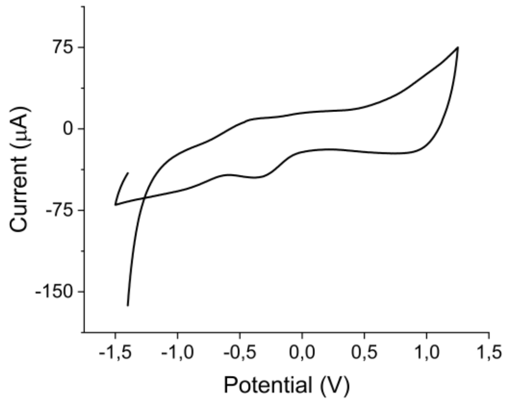


**a**

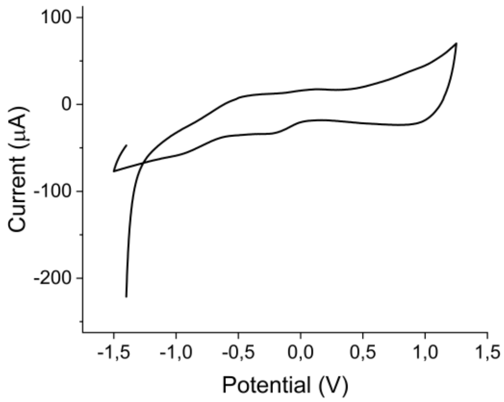


**b**

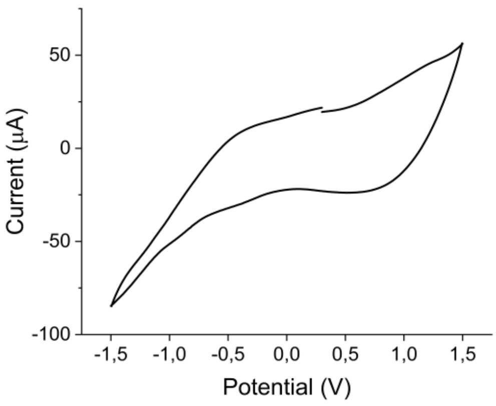


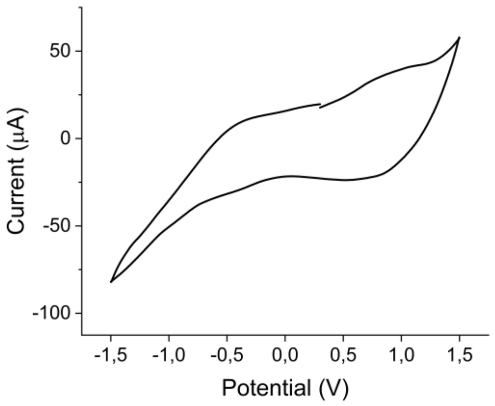

c

d

**Table S1**: First CV scans of 1 mmol/L **C1** (**a**) and **C2** (**b**) solutions in DCM:DMF 9:1 with 0.1 mol/L $Bu_4NPF_6$ as the supporting electrolyte were recorded at a scan rate of 20 mV/s and a temperature of 20°C; cyclic voltammetry scans starting at the equilibrium potential (open current) of **C1** (**c**) and **C2** (**d**).

Clark-type electrode original profiles

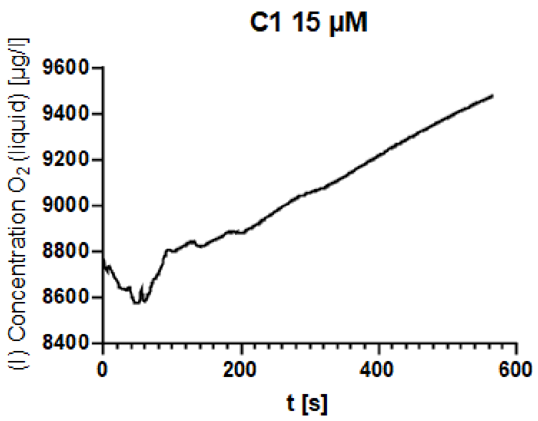


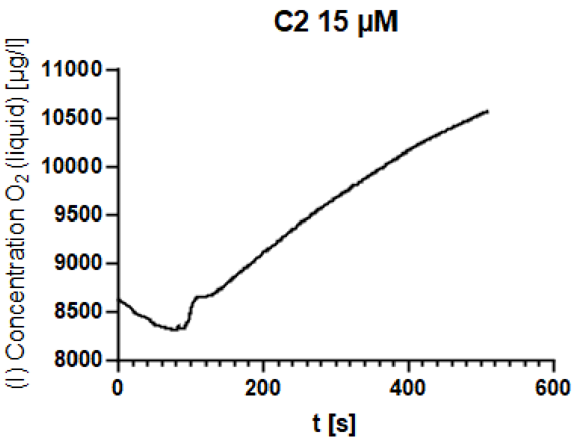


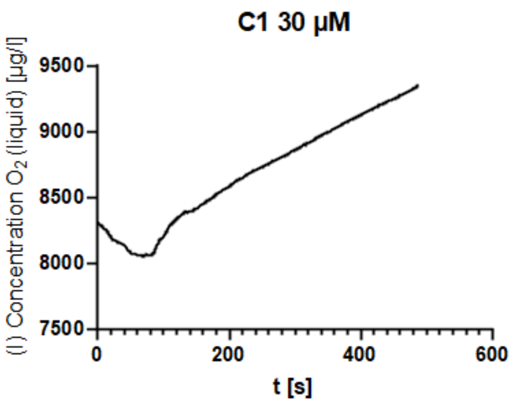


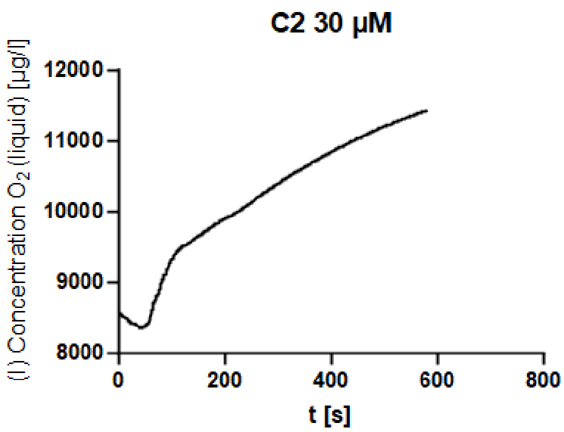


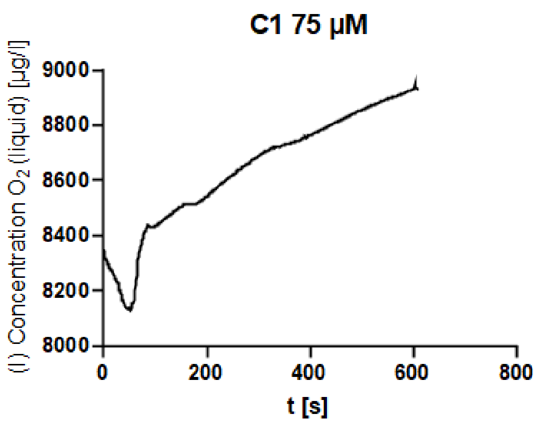


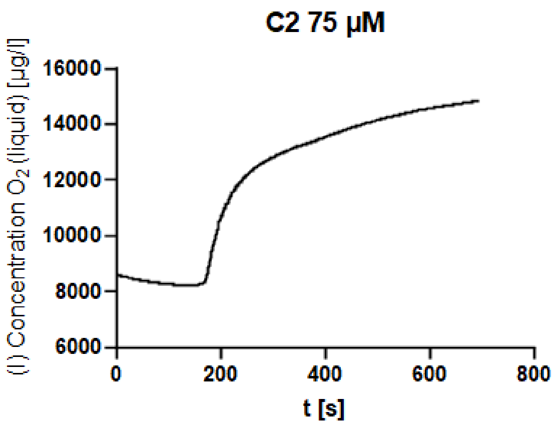

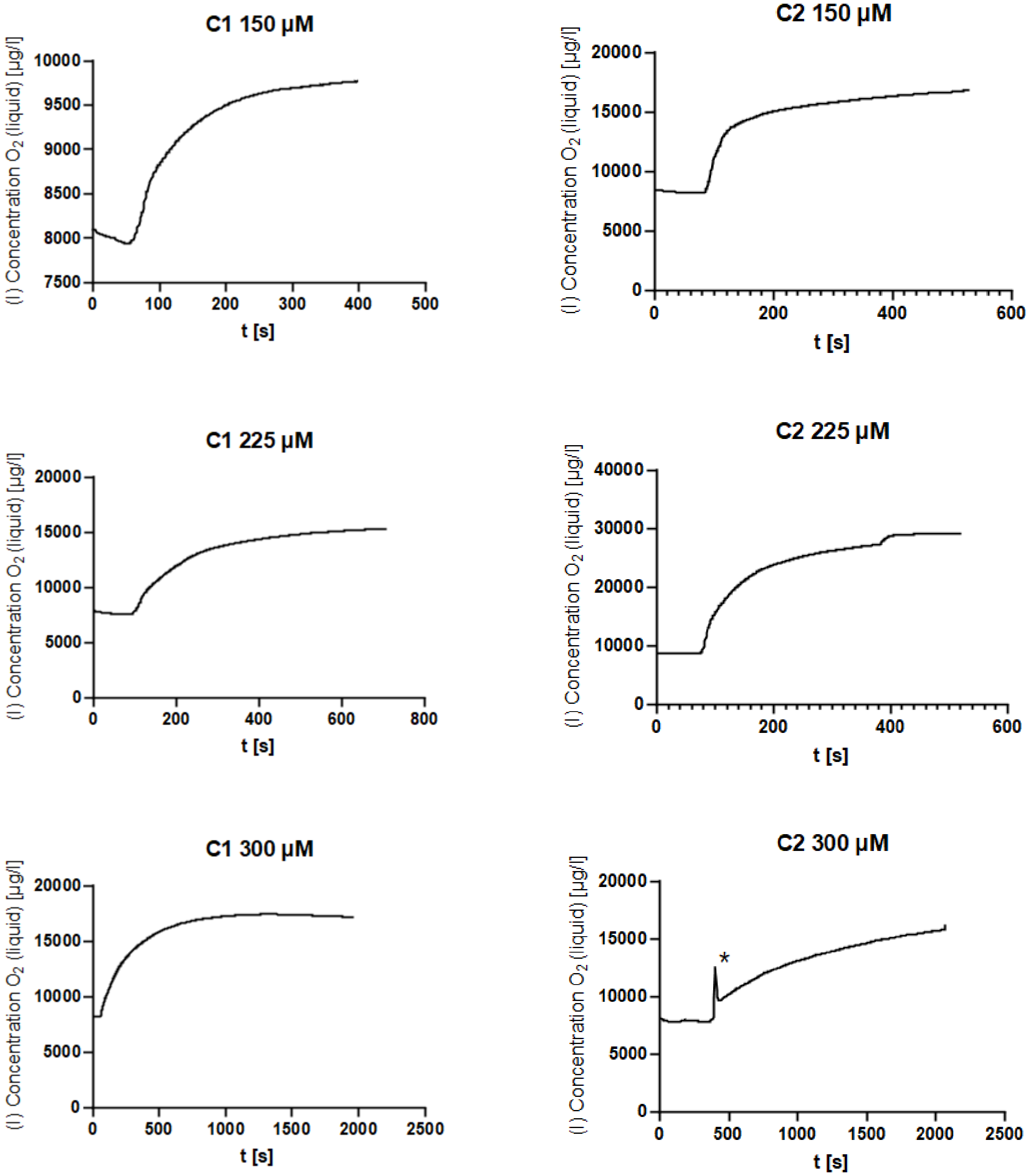


**Table S2**: Original concentration to time report obtained by Clark-type oximeter analysis. ***C2** 300 µmol/L profile shows an unusual peak around the 500th second: this is due to a incorrect mixing procedure; in this case the curve was extrapolated and $V_{max}$ obtained as tangent of the newly obtained curve.

## Determination of proton dissociation constants

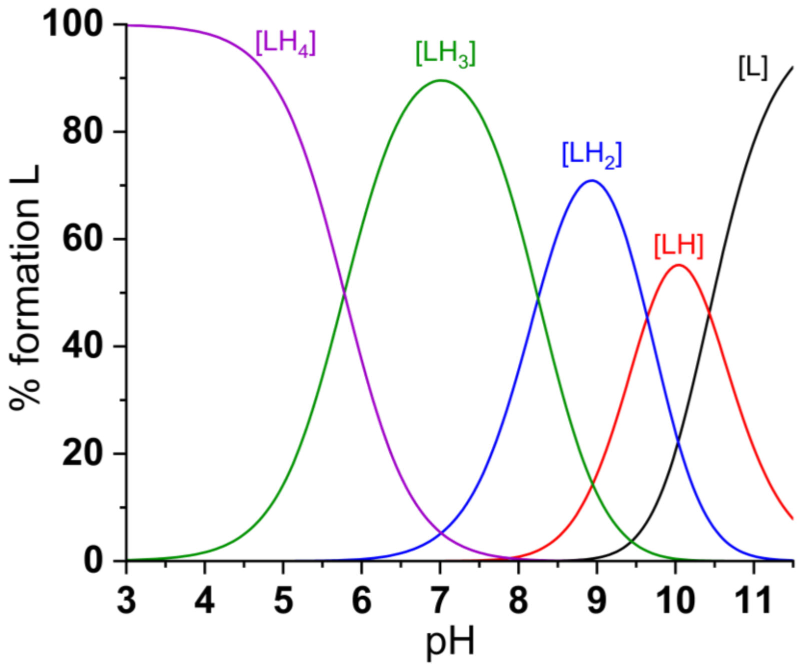


**Figure S19**: Representative distribution diagram of **L1** in aqueous solution (p$K_a$ values obtained by potentiometry, 0.3 mmol/L, I = 0.1 mol/L KCl, T = 298.2 K).

XRD data

| | |
|---|---|
| **Crystal Data** | |
| Formula | C17 H22 N2 O2, C H4 O |
| Formula Weight | 318.41 |
| Crystal System | monoclinic |
| Space group | P21/c (No. 14) |
| a, b, c [Angstrom] | 11.524(1) 8.0646(6) 20.061(2) |
| alpha, beta, gamma [deg] | 90 106.586(6) 90 |
| V [Ang**3] | 1786.9(3) |
| Z | 4 |
| D(calc) [g/cm**3] | 1.184 |
| Mu(CuKa) [ /mm ] | 0.648 |
| F(000) | 688 |
| Crystal Size [mm] | 0.08 x 0.02 x 0.02 |
| **Data Collection** | |
| Temperature (K) | 200 |
| Radiation [Angstrom] | CuKa 1.54178 |
| Theta Min-Max [Deg] | 2.3, 72.3 |

| | |
|---|---|
| Dataset | -14: 14 ; -9: 8 ; -24: 24 |
| Tot., Uniq. Data, R(int) | 22178, 3636, 0.074 |
| Observed Data [I > 0.0 sigma(I)] | 2853 |
| **Refinement** | |
| Nref, Npar | 3636, 223 |
| R, wR2, S | 0.0518, 0.1432, 1.07 |
| Min. and Max. Resd. Dens. [e/Ang^3] | -0.25, 0.37 |
| **CCDC deposition number:** | **2367089** |

**Table S3**. Crystallographic data of **L1** structure.

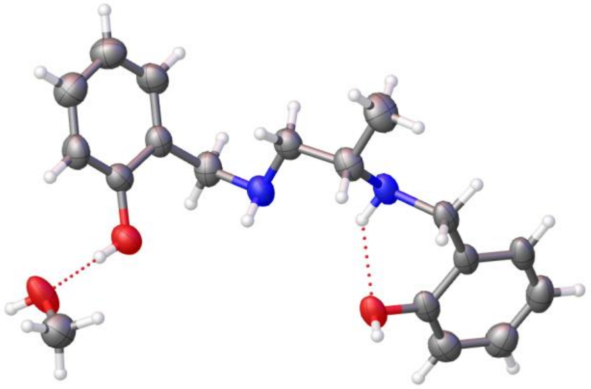

**Figure S20:** ORTEP representation of the asymmetric unit of compound **L1**.$CH_3OH$. With exception of the hydrogen atoms, which are drawn as spheres of arbitrary radius, all other atoms are represented as thermal displacement ellipsoids of 50% probability level. Highlighted in red the two most significant hydrogen bonds that determine the conformation of the molecule and the interaction with the methanol.

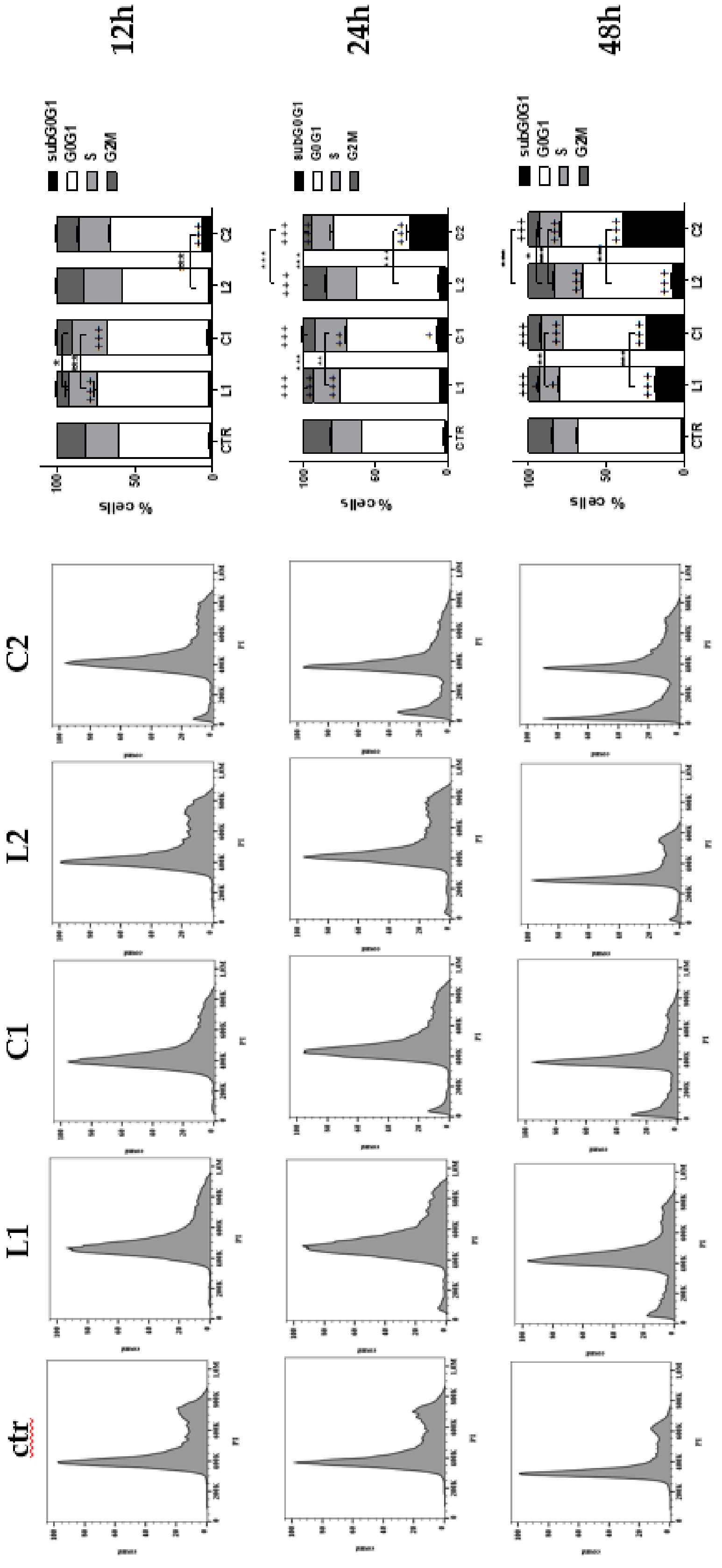


**Figure S21**: Monoparametric DNA analysis of cellular cycle distribution after 12-48h treatment with compounds. Three distinct phases could be recognized in the proliferating cell population, corresponding to different peaks: G0/G1, S and G2/M phase. Values are mean ± SD of three separate experiments, each carried out in triplicate. Statistical significance is indicated as follows: $^{+++}$p<0.001, $^{++}$ p<0.01, +p<0.05 values vs controllo; *p<0.05, **p<0.01, ***p<0.001 C vs L.
